# Three *Prochlorococcus* Cyanophage Genomes: Signature Features and Ecological Interpretations

**DOI:** 10.1371/journal.pbio.0030144

**Published:** 2005-04-19

**Authors:** Matthew B Sullivan, Maureen L Coleman, Peter Weigele, Forest Rohwer, Sallie W Chisholm

**Affiliations:** **1**Joint Program in Biological Oceanography, Woods Hole Oceanographic Institution and Massachusetts Institute of TechnologyCambridge, MassachusettsUnited States of America; **2**Department of Civil and Environmental Engineering, Massachusetts Institute of TechnologyCambridge, MassachusettsUnited States of America; **3**Department of Biology, Massachusetts Institute of TechnologyCambridge, MassachusettsUnited States of America; **4**Department of Biology, San Diego State UniversitySan Diego, CaliforniaUnited States of America; University of ArizonaUnited States of America

## Abstract

The oceanic cyanobacteria *Prochlorococcus* are globally important, ecologically diverse primary producers. It is thought that their viruses (phages) mediate population sizes and affect the evolutionary trajectories of their hosts. Here we present an analysis of genomes from three *Prochlorococcus* phages: a podovirus and two myoviruses. The morphology, overall genome features, and gene content of these phages suggest that they are quite similar to T7-like (P-SSP7) and T4-like (P-SSM2 and P-SSM4) phages. Using the existing phage taxonomic framework as a guideline, we examined genome sequences to establish “core” genes for each phage group. We found the podovirus contained 15 of 26 core T7-like genes and the two myoviruses contained 43 and 42 of 75 core T4-like genes. In addition to these core genes, each genome contains a significant number of “cyanobacterial” genes, i.e., genes with significant best BLAST hits to genes found in cyanobacteria. Some of these, we speculate, represent “signature” cyanophage genes. For example, all three phage genomes contain photosynthetic genes *(psbA, hliP)* that are thought to help maintain host photosynthetic activity during infection, as well as an aldolase family gene *(talC)* that could facilitate alternative routes of carbon metabolism during infection. The podovirus genome also contains an integrase gene *(int)* and other features that suggest it is capable of integrating into its host. If indeed it is, this would be unprecedented among cultured T7-like phages or marine cyanophages and would have significant evolutionary and ecological implications for phage and host. Further, both myoviruses contain phosphate-inducible genes *(phoH* and *pstS)* that are likely to be important for phage and host responses to phosphate stress, a commonly limiting nutrient in marine systems. Thus, these marine cyanophages appear to be variations of two well-known phages—T7 and T4—but contain genes that, if functional, reflect adaptations for infection of photosynthetic hosts in low-nutrient oceanic environments.

## Abstract 2

An analysis of the genome sequences of three phages capable of infecting marine unicellular cyanobacteria Prochlorococcus reveals they are genetically complex with intriguing adaptations related to their oceanic environment

## Introduction

*Prochlorococcus* is the numerically dominant primary producer in the temperate and tropical surface oceans [1]. These cyanobacteria are the smallest known photosynthetic organisms (less than a micron in diameter), yet are significant contributors to global photosynthesis [2,3] because they occur in high abundance (as many as 10^5^ cells/ml) throughout much of the world's oceans. They are adapted to living in low-nutrient oceanic regions [4] and are physiologically and genetically diverse with at least two “ecotypes” that have distinctive light physiology [5], nitrogen [6] and phosphorus (L. R. Moore, personal communication) utilization, and copper [7] and virus (phage) [8] sensitivity. Cyanobacterial phages are also abundant in these environments [8,9,10,11,12] and have a small, but significant, role in mediating population sizes [9,10]. Further, cyanophages likely play a role in maintaining the extensive microdiversity within marine cyanobacteria [9,10] through keeping “competitive dominants” (sensu [13]) in check, as well as by carrying photosynthetic “host” genes [14,15,16] and mediating horizontal transfer of genetic material between cyanobacterial hosts [14].

Although there are more than 430 completed double-stranded DNA phage genomes in GenBank, only nine phages with published genomes infect marine hosts (cyanophage P60; vibriophages VpV262, KVP40, VP16T, VP16C, K139, and VHML; roseophage SIO1; and *Pseudoalteromonas* phage PM2). Of those nine, only one infects cyanobacteria (cyanophage P60, a member of the Podoviridae). P60 was isolated from estuarine waters using *Synechococcus* WH7803 as a host and appears most closely related to the T7-like phages [17]. It contains 11 T7-like phage genes and has no genes with homology to non-T7-like phages. However, it lacks the conserved T7-like genome architecture. Thus, P60 is thought to be only distantly related to the T7-like phages, but still part of a T7 supergroup [18] proposed by Hardies et al. [19]. The T7 supergroup also contains two other marine phages (roseophage SIO1 and vibriophage VpV262) that show similarity to some (three) T7-like genes. However, these phages lack many T7-like genes including the hallmark T7-like RNA polymerase (RNAP) gene [18]. Thus, there is clearly a gradient in relatedness among the T7 supergroup, with these newer marine phage genomes at the distant, less-similar end of the group.

Marine phages are subject to different selection pressures (e.g., dispersal strategies, encounter rates, limiting nutrients, and environmental variability) than their relatively well-studied terrestrial counterparts. Thus, beyond informing phage taxonomy, the analysis of their genomes should unveil “signatures” of these selective agents. For example, genomic analysis of two marine phages, roseophage SIO1 [20] and vibriophage KVP40 [21], has revealed phosphate-inducible genes. It is thought that these genes play an important regulatory role in the phosphorus-limited waters from which they were isolated. Similarly, some *Prochlorococcus* and *Synechococcus* phages (including the three cyanophage genomes presented here) contain core photosynthetic genes that are full-length, conserved, and cyanobacterial in origin [14,15,16]. They are hypothesized to be important for maintaining active photosynthetic reaction centers—and hence the flow of energy—during phage infection [14,15,16].

With a large collection of phages from which to choose [8], we used host range and phage morphology to select strains for sequencing. The selected podovirus (P-SSP7) is very host-specific, infecting a single high-light-adapted (HL) *Prochlorococcus* strain of 21 *Prochlorococcus* and *Synechococcus* strains tested. In contrast, the two myoviruses that were selected cross-infect between *Prochlorococcus* (but not *Synechococcus*) hosts: P-SSM2 can infect three low-light-adapted (LL) host strains, and P-SSM4 can infect two HL and two LL hosts [8]. We had no prior knowledge of the gene content of these phages; thus, with regard to their genomes, these phages were selected randomly.

As mentioned earlier, our first survey of these phage genomes led to the surprising discovery of photosynthetic genes in all three *Prochlorococcus* phages [14], similar to the findings in *Synechococcus* cyanophages [15,16,22]. In this report, we present a more thorough analysis of these three cyanophage genomes, which, we argue, appear to be T7-like (P-SSP7) and T4-like (P-SSM2 and P-SSM4) phages.

## Results/Discussion

### General Features of the Podovirus P-SSP7

**Figure 1:**
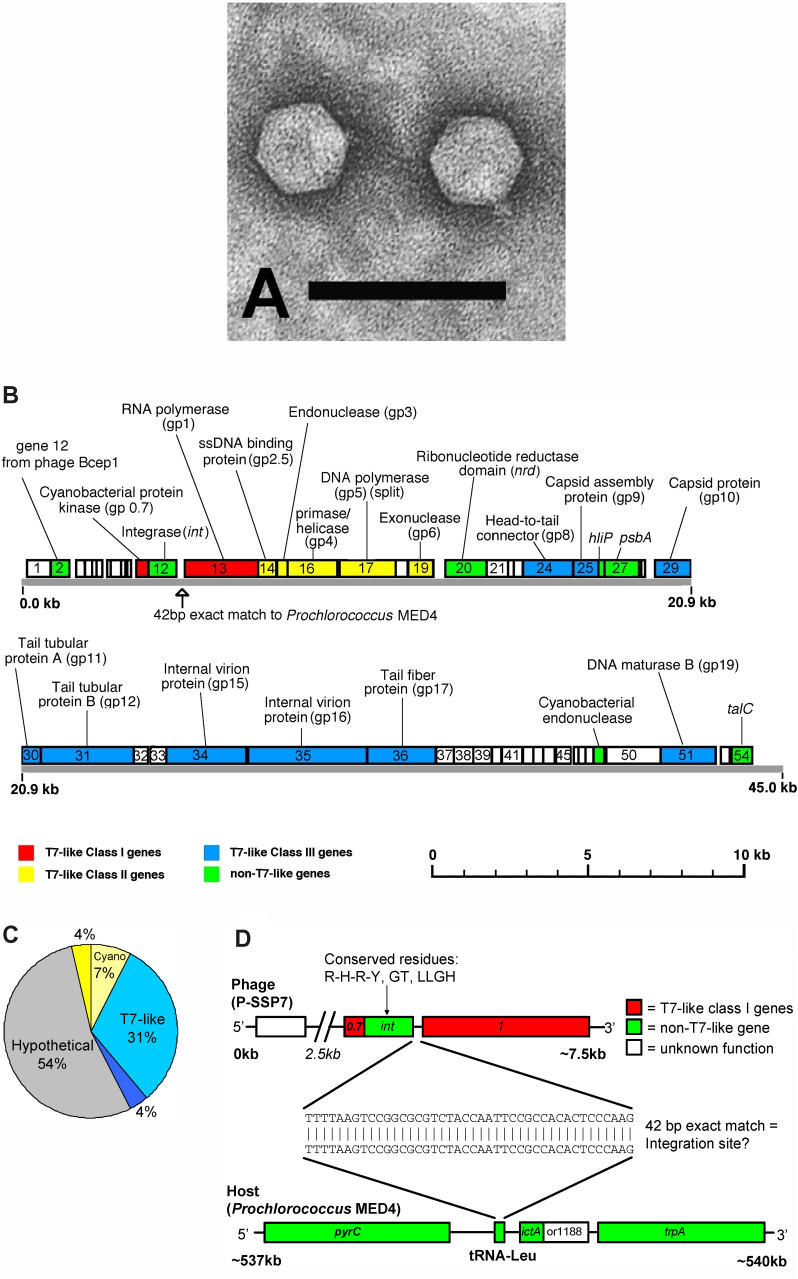
Features of the *Prochlorococcus* Podovirus P-SSP7 (A) Electron micrograph of negative-stained podovirus P-SSP7. Note the distinct T7-like capsid and tail structure. Scale bar indicates 100 nm. (B) Genome arrangement of *Prochlorococcus* podovirus P-SSP7. The ORFs are sequentially numbered within the boxes, and gene names are designated above the boxes. Gene designations use T7 nomenclature for T7-like genes [24] or microbial nomenclature for non-phage genes. Class I, II, and III genes refer to those in T7 [66] that belong to gene regions primarily involved in host transcription of phage genes (class I), DNA replication (class II), and the formation of the virion structure (class III). The ORFs are designated by boxes, and in this genome, all ORFs are oriented in the same direction. Although the phage genome is one molecule of DNA, the representation is broken to fit on a single page. Note that the P-SSP7 genome is most similar to genomes of the T7-like phages. (C) Taxonomy of best BLASTp hits for P-SSP7. Each predicted coding sequence from the phage genomes was used as a query against the nonredundant database to identify the taxon of the best hit (details in Materials and Methods). Blue slices indicate phage hits, while yellow slices indicate cellular hits. (D) Diagrammatic representation of the genomic regions surrounding a putative phage and host integration site. This site consists of a 42-bp exact match between the podovirus P-SSP7 and its host *Prochlorococcus* MED4 located directly downstream of the phage integrase gene and the noncoding strand of a host tRNA gene.

P-SSP7 is morphologically similar to the Podoviridae (tails are short and noncontractile; Figure 1A). It also includes a rectangular region of electron transparency (Figure 1A) that is similar to the gp14/gp15/gp16 core located at the unique portal vertex found in coliphage T7 [23]. Its genome contains 44,970 bp (54 open reading frames [ORFs]; 38.7% G+C content; Figure 1B), including a T7-like RNAP and a phage-related integrase gene (a more detailed analysis of this feature is discussed later). Thus, the P-SSP7 genome is more T7-like or P22-like than φ29-like among the Podoviridae (Table 1). Thirty-five percent of the translated ORFs have best hits to phage proteins; nearly all of these are T7-like, whereas none are P22-like (Figure 1C). Together, these data suggest that P-SSP7 is most closely related to the T7-like phages. Surprisingly, 11% of the translated ORFs have best hits to bacterial proteins, with well over half of these being cyanobacterial (see later discussion). Roughly half (54%) of the translated ORFs could not be assigned a function (Figure 1C).

**Table 1:**
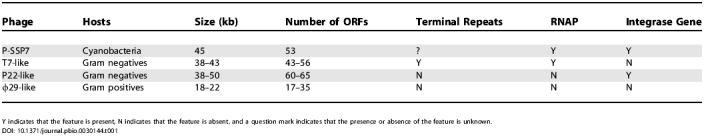
Genome-Wide Characteristics of the *Prochlorococcus* Cyanophage P-SSP7 Relative to the Other Recognized Phage Groups within the Podoviridae [105]

Y indicates that the feature is present, N indicates that the feature is absent, and a question mark indicates that the presence or absence of the feature is unknown

An examination of the genomes of coliphage T7 and its closest coliphage relatives (T3, gh-1, ΦYe03–12, ΦA1122) revealed that they share 26 genes, which we define as core genes (Table 2). P-SSP7 has 15 of these 26 core genes and an additional gene *(0.7)* that is common, but not universal, among T7-like phages (Table 2). Further, only two non-T7-like phage genes were identified in this genome: hypothetical gene *12* from a *Burkholderia* phage, *Bcep1,* of the Myoviridae family, and the phage-related integrase gene discussed later. Strikingly, the T7-like genes found in P-SSP7 are arranged in exactly the same order as in other T7-like phages (Figure 1B). The gene content and genome architecture of P-SSP7 contrast with those from the three other sequenced marine podovirus genomes in the T7 supergroup [17,19,20]. SIO1 and VpV262 lack the hallmark T7-like RNAP and contain only three T7-like core genes (Table 2), whereas cyanophage P60 contains 11 core genes (Table 2) but clearly lacks the conserved T7-like genome architecture [17].

**Table 2:**
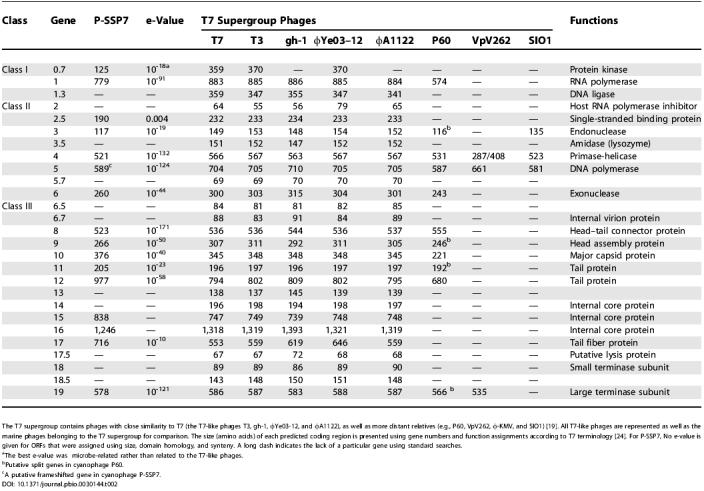
Shared Genes in T7-Like Phages

The T7 supergroup contains phages with close similarity to T7 (the T7-like phages T3, gh-1, φYe03-12, and φA1122), as well as more distant relatives (e.g., P60, VpV262, φ-KMV, and SIO1) [19]. All T7-like phages are represented as well as the marine phages belonging to the T7 supergroup for comparison. The size (amino acids) of each predicted coding region is presented using gene numbers and function assignments according to T7 terminology [24]. For P-SSP7, No e-value is given for ORFs that were assigned using size, domain homology, and synteny. A long dash indicates the lack of a particular gene using standard searches

^a^The best e-value was microbe-related rather than related to the T7-like phages

^b^Putative split genes in cyanophage P60

^c^A putative frameshifted gene in cyanophage P-SSP7

The putative functions of the 16 T7-like genes in P-SSP7 would allow for the majority of host interactions and phage production as follows (T7-like gene designations are shown in parentheses): shutdown of host transcription *(0.7),* phage gene transcription *(1),* degradation of host DNA *(3, 6),* DNA replication *(1, 2.5, 4, 5),* formation of a channel across the cell envelope via an extensible tail *(15, 16)* [24], DNA packaging *(19),* and virion formation *(8, 9, 10, 11, 12, 17).* We found two stretches of DNA (frame +1 from nucleotides 9994–10525, then frame +3 from nucleotides 10485–11759) with matches to T7 gp5 (DNA polymerase [DNAP]): one corresponding to the 3′-exonuclease and one to the polymerase (nucleotidyl transferase) segments of the T7 enzyme. This region may encode a split variant of T7 family DNAP (V. Petrov and J. Karam, personal communication), an arrangement that has been shown to be functional in archaea [25] and some T4-like phages (V. Petrov and J. Karam, personal communication).

As described earlier, we identified only 15 of the 26 core T7-like genes in P-SSP7. What are the functions of the absent gene set? It includes genes that in T7 are involved in ligation of DNA fragments *(1.3),* inhibition of host RNAP *(2),* interactions that are specific to the host cell envelope during virion formation *(6.7, 13, 14),* lysis events *(3.5, 17.5),* small-subunit terminase activity *(18),* and unknown functions *(5.7, 6.5, 18.5)* [23]. These same genes are also absent in the marine podovirus genomes in the T7 supergroup (cyanophage P60, vibriophage VpV262, and roseophage SIO1; Table 3). If we assume a conserved genomic architecture among the T7-like phages, we find hypothetical ORFs in homologous positions to these T7 core genes in P-SSP7 (Figure 1B) that may fulfill these core (e.g., *5.7, 6.5, 6.7, 13, 14, 17.5, 18, 18.5*) and common (e.g., antirestriction gene *0.3*) T7-like gene functions. Alternatively, their functions may be unnecessary for this phage.

**Table 3:**
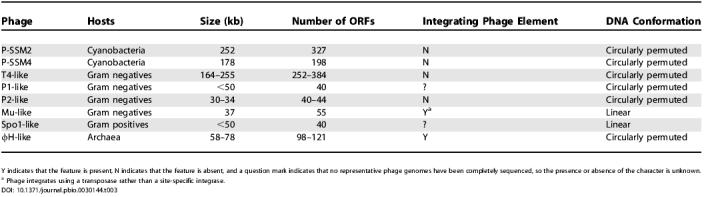
Genome-Wide Characteristics of the *Prochlorococcus* Cyanomyophages P-SSM2 and P-SSM4 Relative to the Other Recognized Phage Groups within the Myoviridae [105]

Y indicates that the feature is present, N indicates that the feature is absent, and a question mark indicates that no representative phage genomes have been completely sequenced, so the presence or absence of the character is unknown

^a^ Phage integrates using a transposase rather than a site-specific integrase

The P-SSP7 genome assembled as a circular chromosome, suggesting that it is circularly permuted, thus lacking the terminal repeats that are common among T7-like phages [26]. Confirmation of this hypothesis would require direct sequencing of the genome ends (I. Molineux, personal communication), which was not possible in this study because of the difficulty of obtaining significant quantities of purified DNA [27].

### Hypothesized Lysogeny in P-SSP7

One of the more interesting discoveries in the podovirus genome is the presence of a tyrosine site-specific recombinase *(int)* gene (Figure 1B), which in temperate phages encodes a protein that enables the phage to integrate its genome into the host genome [28]. T7 is a classically lytic phage, and there has been only one other report of *int* genes in a T7-like phage: in an integrated prophage in the Pseudomonas putida KT2440 genome [29]. The P-SSP7 *int* contains conserved amino acid motifs previously identified for site-specific recombinases (Arg-His-Arg-Tyr, Leu-Leu-Gly-His, and Gly-Thr [30]) suggesting it is functional. Downstream of *int,* we find a 42-bp sequence that is identical to part of the noncoding strand of the leucine tRNA gene in the phage's host genome (*Prochlorococcus* MED4) (Figure 1D). tRNA genes are a common integration site for phages and other mobile elements [31], adding support to the hypothesis that this *int* gene is functional.

P-SSP7 was isolated from surface ocean waters at the end of summer stratification [8], when nutrients are extremely limiting. We have hypothesized [8] that the integrating phase of the temperate-phage life cycle may be selected for under these conditions; thus, finding the *int* gene in this particular phage is consistent with this hypothesis. None of the complete genome sequences of cyanobacterial hosts reported to date have intact prophages [4,32,33,34]. Moreover, temperate phages have not been induced from unicellular freshwater or marine cyanobacterial cultures [9,35,36]. Although some field experiments suggest that temperate cyanophages can be induced from *Synechococcus* [37,38], prophage integration has not been demonstrated. Thus, experimental validation that P-SSP7 is capable of integration would confirm indirect evidence and establish a valuable experimental system.

### General Features of the Myoviruses P-SSM2 and P-SSM4

**Figure 2:**
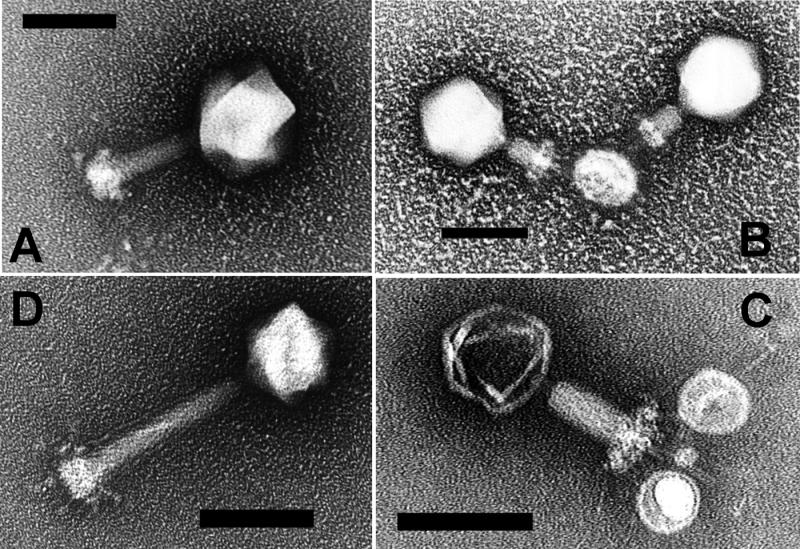
Electron Micrograph of Negative-Stained *Prochlorococcus* Myoviruses P-SSM2 and P-SSM4 Myovirus P-SSM2 with (A) non-contracted tail and (B) contracted tail, and myovirus P-SSM4 with (C) contracted tail and (D) non-contracted tail. Note the T4-like capsid, baseplate, and tail structure in both myoviruses. Scale bars indicate 100 nm.

**Figure 3:**
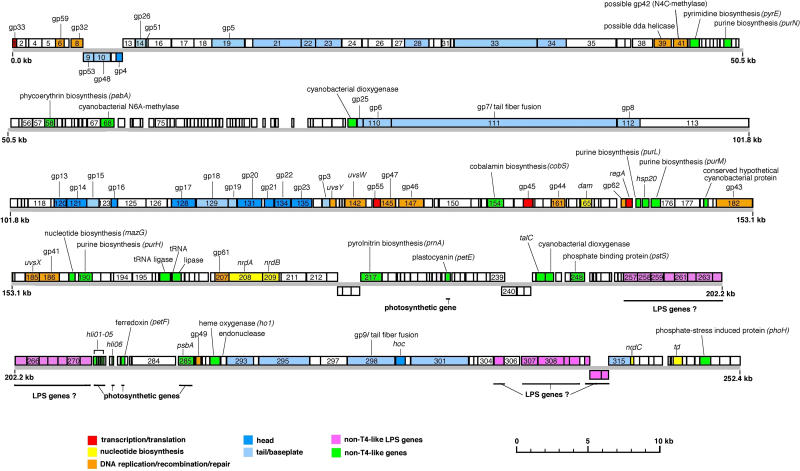
Genome Arrangement of the *Prochlorococcus* Myovirus P-SSM2 Gene names are designated above the box representing the ORF where genes were identified; descriptions of genes are in Table 4. The ORFs located above the centering line are on the forward DNA strand, whereas those below the line are on the reverse strand. Although the genome is one molecule, the representation is broken to fit the page. Colors indicate the putative role for the identified genes as inferred from T4 phage. Gene designations use T4 nomenclature for T4-like genes [104] or microbial nomenclature for non-phage genes.

**Figure 4:**
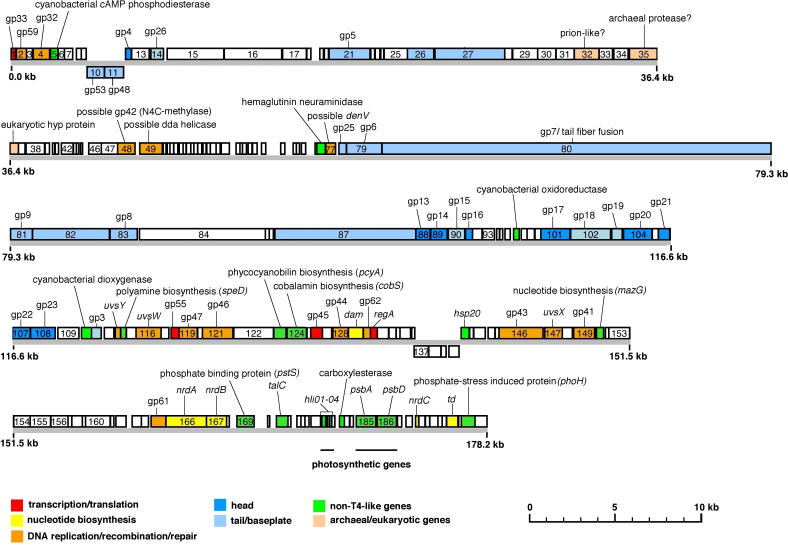
Genome Arrangement of the *Prochlorococcus*Myovirus P-SSM4 Gene nomenclature is as in Figure 3.

**Figure 5:**
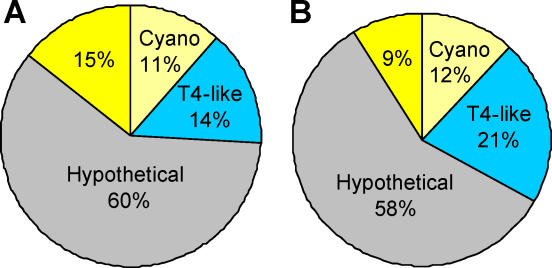
Taxonomy of Best BLASTp Hits for P-SSM2 and P-SSM4 Each predicted coding sequence from both phage genomes was used as a query against the nonredundant database to identify the taxon of the best hit (details in Materials and Methods). Blue slices indicate phage hits, while yellow slices indicate cellular hits.

P-SSM2 and P-SSM4 are morphologically similar to the Myoviridae (tails are long and contractile; Figure 2). Both have an isometric head, contractile tail, baseplate, and tail fiber structures (Figure 2) that are most consistent (but see isometric head discussion later) with the morphological characteristics of the T4-like phages [39]. Their genomes also have general characteristics that are fully consistent with T4-like status within the Myoviridae (Table 3). Both genomes are relatively large: P-SSM2 has 252,401 bp (327 ORFs; 35.5% G+C content; Figure 3) and P-SSM4 has 178,249 bp (198 ORFs; 36.7% G+C content; Figure 4). An apparent strand bias is noteworthy because only 12 (of 327) and six (of 198) ORFs are predicted on the minus strand in the P-SSM2 and P-SSM4 genomes, respectively. Similar to the lytic T4-like phages, integrase genes were absent. Both genomes assembled and closed, suggesting the circularly permuted chromosome common among the T4-like phages (Table 3). A large portion of the nonhypothetical ORFs have best hits to phage proteins (14% and 21%, respectively) and bacterial proteins (26% and 21%, respectively; Figure 5). The phage hits were most similar to T4-like phage proteins, and about half of the bacterial ORFs were most similar to those from cyanobacteria. As with P-SSP7, most of the translated ORFs from P-SSM2 and P-SSM4 could not be assigned a function (60% and 58%, respectively). The majority of the differences between these two phages are due to the presence of two large clusters of genes (24 total) in P-SSM2 (see Figure 3) that are absent from P-SSM4. These clusters contain many sugar epimerase, transferase, and synthase genes that we hypothesize to be involved in lipopolysaccharide (LPS) biosynthesis. The large genome size, collective gene complement, and morphology suggest both P-SSM2 and P-SSM4 are most closely related to T4-like phages.

**Table 4:**
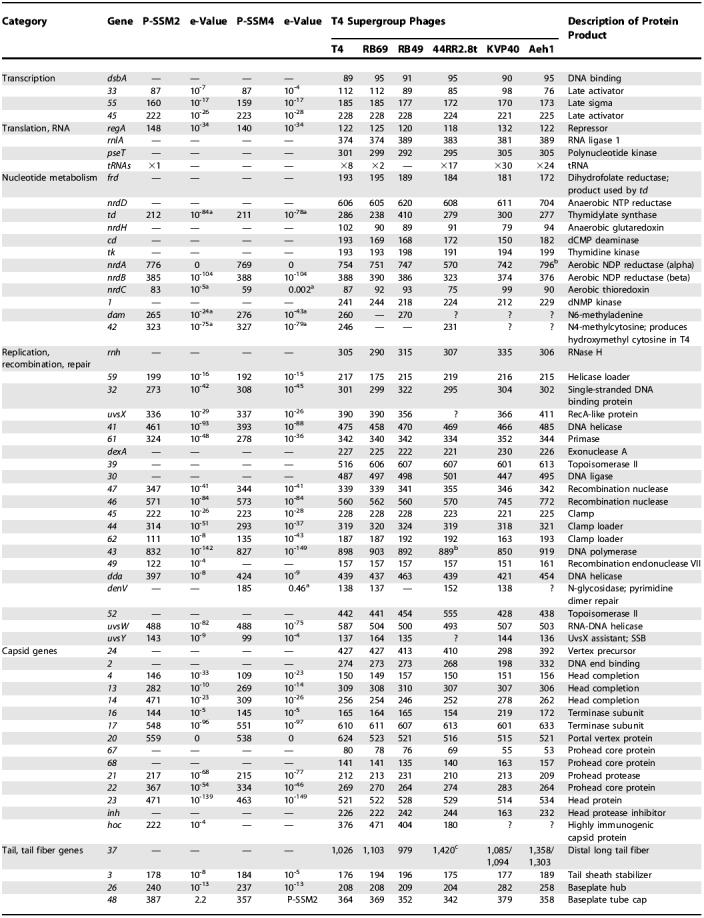
Shared Genes in T4-like Phages

**Figure 6:**
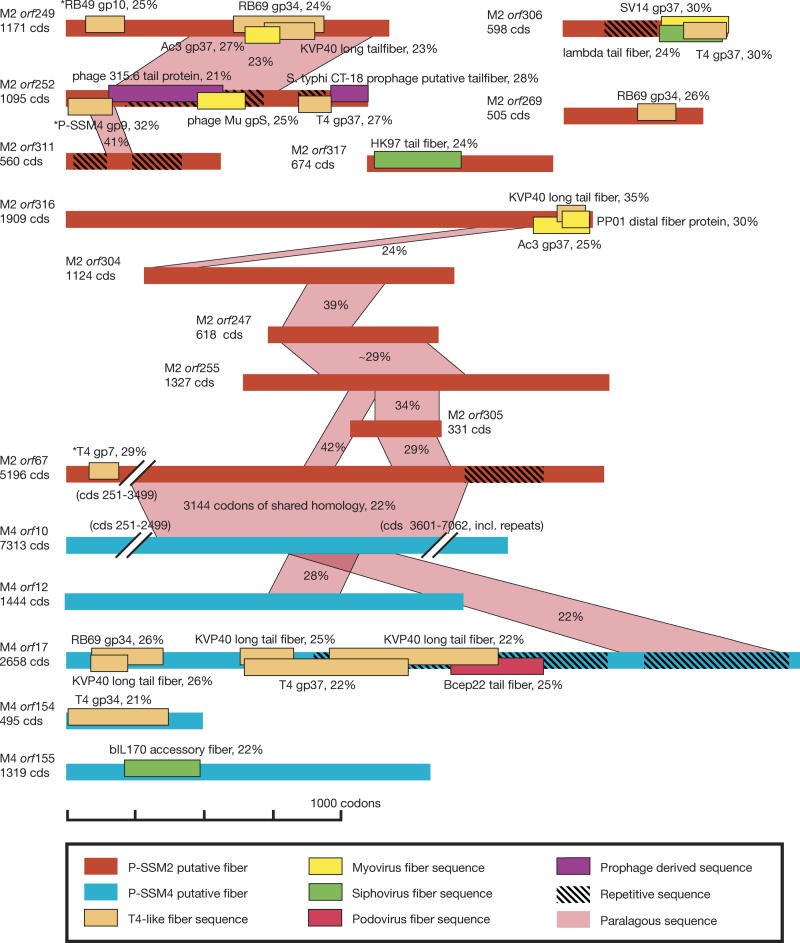
Bioinformatically Identified Tail Fiber Genes from *Prochlorococcus* Myoviruses Red bars indicate P-SSM2 ORFs (labeled as M2); blue bars indicate P-SSM4 ORFs (labeled as M4). Due to space constraints, P-SSM2 orf67 and P-SSM4 orf10 are broken as indicated.

Table modified from [22,104]. The T4 supergroup is divided into T-evens (e.g., T4 and RB69), pseudo T-evens (e.g., RB49 and 44RR2.8t), Schizo T-evens (e.g., Aeh1), and the Exo T-evens (e.g., S-PM2) [106,107]. For previously published T4 supergroup phages, only the size (amino acids) of selected predicted coding regions are presented using gene names according to T4 terminology. For P-SSM2 and P-SSM4, the size of each translated gene and the e-value of the best phage–T4-like (or microbe-related see below) e-value is presented; Where no e-value is given, these ORFs were assigned based upon size, domain homology, and synteny except where “Fig.6” is listed, which refers to designations made using tail fiber analyses summarized in Figure 6, and P-SSM2 or P-SSM4 indicates designation made through paralogy. A long dash indicates the lack of a particular gene

^a^The best e-value was microbe-related rather than related to T4-like phages

^b^The gene is split into two segments, often by an intron or homing endonuclease

^c^The gene is fused

**Table 4:**
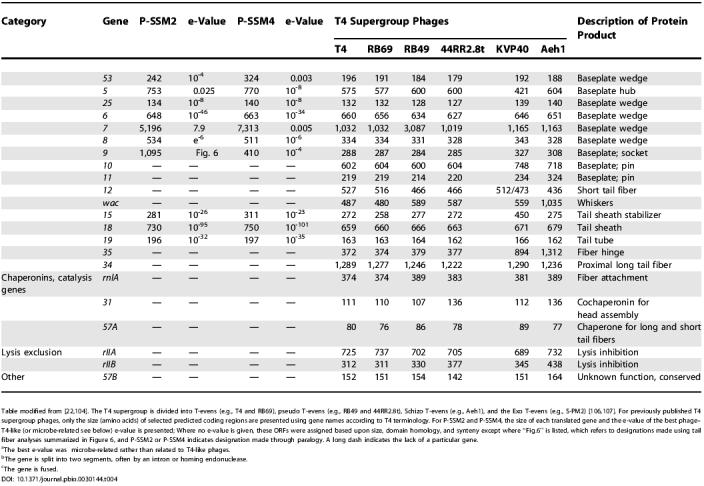
Continued

The six sequenced T4-like phage genomes (T4, RB69, RB49, 44RR2.8t, KVP40, and Aeh1; available as of 15 May 2004 at http://phage.bioc.tulane.edu/) share 75 genes (Table 4), which suggests a core gene complement required for T4-like phage infection. This core contains 18 genes involved in DNA replication, recombination, and repair, seven regulatory genes, ten nucleotide metabolism genes, 34 virion structure and assembly genes, and six genes involved in chaperonin, lysis exclusion, and other activities. Again, despite cyanobacterial hosts being quite divergent from the hosts of these other T4-like phages, our myoviruses contained 43 and 42 of the 75 T4-like core genes, as well as other noncore T4-like genes in each phage (*uvsX, uvsY,* and possibly *dam, 42,* and *hoc* in P-SSM2; *uvsX, uvsY,* and possibly *dam, 42,* and *denV* in P-SSM4; Table 4). Furthermore, aside from the low-complexity tail fiber related genes (see “Tail-Fiber-Related Genes in the Myoviruses” below), we found no genes with sequence similarity to any phage type other than T4-like phages.

Slightly fewer than half of the core T4-like genes were absent in both myoviruses P-SSM2 and P-SSM4. P-SSM2 and P-SSM4 lack the genes required for anaerobic nucleotide biosynthesis *(nrdD*, *nrdG*, and *nrdH),* which is perhaps not surprising because these phages were isolated from the well-mixed, oxygenated surface oceans. Both myoviruses also lack homologs to the prohead core-encoding genes *(67* and*68)* of the T4-like phages (Table 4). However, we note that the capsids of both *Prochlorococcus* myoviruses are isometric (see Figure 2), rather than prolate as is often observed for other T4-like phage capsids [39]. In T4, mutations in the prohead core proteins (gp67 and gp68) are known to cause a capsid structural defect whereby isometric heads are observed [40,41,42]. Thus, functional homologs of prohead core proteins may not be required for the formation of isometric heads in these *Prochlorococcus* myoviruses.

Other T4-like phage gene functions may be represented by divergent homologs filling the T4-like phage role in these cyanomyophages. P-SSM2 and P-SSM4 lack core T4-like chaperonin genes (*rnlA, 31,* and *57A;* Table 4) and nucleotide metabolism genes (T4-like pyrimidine biosynthesis: *cd, frd, 1,* and *tk*; Table 4). However, both P-SSM2 and P-SSM4 contain non-T4-like hsp20-family chaperonins, as well as a non-T4-like gene *(mazG)* that in bacteria is involved in degradation of DNA (Table 5) [43,44]. Furthermore, P-SSM2 contains ORFs with high sequence similarity to host-encoded homologs of five genes involved in pyrimidine *(pyrE)* and purine *(purH, purL, purM,* and *purN)* biosynthesis (Table 5). These non-T4-like genes might compensate for T4-like nucleotide metabolism and/or chaperone genes that are absent. Despite the structural similarities between our myophages (see Figure 2) and the T4-like phages, some core virion structural genes (e.g., head genes, *2, 24, 67, 68,* and *inh*; tail/tail fiber genes, *10, 11, 12, 34, 35, 37,* and *wac*) have yet to be identified in these myophage genomes (see Table 4). Similarly, genes involved in transcriptional regulation *(dsbA, rnlA,* and *pseT),* lysis events *(rIIa* and *rIIb),* and replication, recombination, and repair (DNA ligase, *30;* topoisomerases, *39* and *52;* RNase H, *rnh;* and an exonuclease, *dexA*) also have yet to be identified.

**Table 5:**
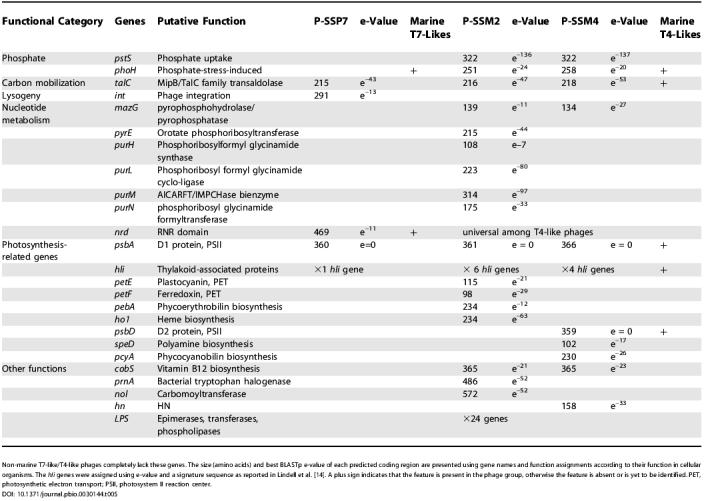
Summary Table of Unique Features of *Prochlorococcus* Cyanophage Genomes That Are Uncommon among Known Phages

Non-marine T7-like/T4-like phages completely lack these genes. The size (amino acids) and best BLASTp e-value of each predicted coding region are presented using gene names and function assignments according to their function in cellular organisms. The *hli* genes were assigned using e-value and a signature sequence as reported in Lindell et al. [14]. A plus sign indicates that the feature is present in the phage group, otherwise the feature is absent or is yet to be identified. PET, photosynthetic electron transport; PSII, photosystem II reaction center

### Tail-Fiber-Related Genes in the Myoviruses

Sequence analysis of phage tail fiber genes has revealed extensive swapping of gene fragments between loci [45,46]. Such exchanges yield phages with altered host ranges [47]. Although this mosaic gene construction makes computational identification of tail fiber genes by sequence homology difficult, we have attempted to do so in the two *Prochlorocococcus* T4-like genomes. The analysis is motivated by the belief that understanding mechanisms of attachment and host range is critical for developing assays for studying phage–host interactions in wild populations—one of the underlying motivations of our work with this system.

We identified ORFs as potential tail fiber genes by a three-tiered bioinformatics approach using sequence similarity, repeat analysis, and paralogy (details in Materials and Methods). First, sequence similarity to known tail fiber genes was used to add ORFs to the pool of possible tail fiber genes (Figure 6). Seven ORFs in P-SSM2 and three ORFs in P-SSM4 had similarity to known tail fiber genes. In T4, the long tail fiber of T4 is composed of four protein subunits including a proximal-end subunit (gp34) anchoring the fiber to the phage baseplate and a distal-end subunit (gp37) responsible for host recognition and attachment (reviewed in [48]). Thus P-SSM2 and P-SSM4 ORFs contained regions similar to T4-like phage distal tail fiber genes (gp37; P-SSM2 orf023, orf033, orf295, and orf298; P-SSM4 orf087) and proximal tail fiber genes (gp34; P-SSM2 orf295 and orf315; P-SSM4 orf026 and orf087). Further, two P-SSM2 ORFs (orf034 and orf315) and a P-SSM4 ORF (orf027) are similar to other known tail fiber genes, albeit with low sequence similarity, and for only a small portion of the ORF.

Second, ORFs containing repeat sequences were added to the pool of possible tail fiber genes. Both simple (amino acid triplets) and complex (longer amino acid motifs) repeats are associated with phage tail fiber genes [49,50]. Simple repeats are found in two P-SSM2 ORFs (orf23 and orf28; Figure 6), with nearly 49% of orf028 encoding the simple triplet repeat Gly-X-Y (where X and Y are often proline, serine, or threonine). Proteins with extended runs of these collagen-like amino acid motifs are thought to fold into trimeric coiled coils, consistent with a tail-fiber-like structure [50]. Complex repeat motifs of 15 to 51 amino acids in length are found in P-SSM2 (orf111 and orf298) and P-SSM4 (orf087; Figure 6). Some of these motifs are similar to those found in the long distal tail fiber (gp37) and short tail fiber (gp12) genes in T4, where they encode tandem, beta-strand-rich, supersecondary structural elements that are correlated with the beaded or knobbed shaft structure of these tail fibers [49,51].

Third, possible tail-fiber-encoding ORFs were identified through paralogy to other *Prochlorococcus* phage tail fiber ORFs already identified (Figure 6). This approach follows the observation of homology between three T4 tail fiber genes (gp12, gp34, and gp37) [49], which are thought to have arisen via gene duplication events [52]. These analyses added four ORFs to the pool of possible tail fiber genes for P-SSM2 (orf021, orf022, orf293, and orf301) and two for P-SSM4 (orf080 and orf082).

After identification of a pool of putative tail fiber genes, we used sequence similarity to known tail fiber and/or baseplate genes as a guideline to annotate ORFs according to the known T4 phage architecture. Three tail-fiber-like ORFs of P-SSM2 (orf111, orf295, and orf298) have N-terminal domains that are similar to T4 baseplate proteins (Figure 6). In T4, the N-terminus of the proximal long tail fiber (gp34) is bound to the baseplate via the baseplate protein gp9 and possibly gp10 [53,54,55]. The N-terminus of P-SSM2 orf298 is similar to the P-SSM4 orf081 (a gp9 homolog by sequence), suggesting that P-SSM2 orf298 could be analogous to a T4 proximal long tail fiber subunit (gp34), albeit fused to the baseplate socket in P-SSM2. Although such a fused protein does not appear to exist for the other myophage, P-SSM4, the adjacent reading frame to orf081 encodes a possible tail fiber ORF with significant similarity to C-terminal stretches of P-SSM2 orf298. Thus, it appears that P-SSM4 orf081 and orf082 are orthologous with the PSSM2 orf298 N- and C-terminal regions, respectively. P-SSM2 orf295 also appears to be a tail fiber fused to a baseplate protein, gp10, which, in T4, may also play a role in binding tail fiber proteins, although this role is less clear. Similarly, the very large homologous genes (>15,000 nt) P-SSM2 orf113 and P-SSM4 orf080 appear fused to baseplate wedge initiator (gp7) homologs, which are not known to bind tail fiber in T4 [53]. Regardless of their precise assignments relative to T4 tail fiber genes, these putative fusions likely encode tail fiber subunits that bind directly to the baseplate through incorporation of their N-termini into the baseplate complex. Assuming that the long tail fibers of P-SSM2 or P-SSM4 are composed of more than one kind of protein subunit, as in T4 [48], we hypothesize that these baseplate-domain-containing tail fibers are unlikely to determine host specificity, but rather are analogous to the proximal long tail fiber (gp34) or short tail fiber (gp12) of T4.

Thus we identify a pool of 12 and five putative tail-fiber-related genes (awaiting experimental confirmation) in the P-SSM2 and P-SSM4 genomes, respectively. Some are quite large relative to those in T4, whereas others appear fused to baseplate genes, which has not been observed for the T4-like phages.

### Metabolic Genes Uncommon among Phages

All three cyanophages contained genes that are not commonly found in phages. We have selected the following cyanobacterial genes for discussion because we hypothesize that they could play defining functional roles in the marine cyanophage–cyanobacterium phage–host system.

#### Photosynthesis-related genes in cyanophages

We previously reported photosynthesis-related genes *(psbA* and *hli)* in all three of these *Prochlorococcus* phages, as well as other photosynthesis genes *(petE, petF,* and *psbD)* in one of the two *Prochlorococcus* myovirus genomes [14]. In addition, genomic analyses have revealed that P-SSM2 contains *pebA* and *ho1,* whereas P-SSM4 contains *pcyA* and *speD* (see Table 5). In cyanobacteria these genes are involved in phycobilin biosynthesis *(ho1*, *pebA,* and *pcyA)* [56,57] and polyamine biosynthesis *(speD)*. Although the phycobilin biosynthesis genes are found in *Prochlorococcus* [4,34], their function is unclear because *Prochlorococcus* does not have the intact phycobilisomes characteristic of most cyanobacteria. These genes are thought to be a remnant of the evolutionary reduction of the phycobilisome-based antenna to a chlorophyll-b-based antenna [4,58,59,60]. Although low levels of phycoerythrin occur in some LL *Prochlorococcus* strains [61], they have, as yet, no known function in the host.

The polyamine biosynthesis gene *speD* found in the phage has a homolog in all of the marine cyanobacteria with complete genome sequences. Although its function has not been confirmed in these organisms, SpeD is known to catalyze the terminal step in polyamine synthesis in other prokaryotes, and polyamines affect the structure and oxygen evolution rate of the photosystem II (PSII) reaction center in higher plants [62]. Therefore, SpeD, if expressed, may play a role in maintaining the host PSII reaction center during phage infection.

#### Nucleotide metabolism genes

The podovirus P-SSP7 contains an ORF (orf20) with a putative ribonucleotide reductase (RNR) domain (see Table 5). In prokaryotes and T4-like phages, RNRs provide the building blocks for DNA synthesis through catalyzing a thioredoxin-mediated reduction of diphosphates (e.g., rNDP → dNDP) during nucleotide metabolism [63]. Among T7-like genomes, these domains have been observed only in marine phages (see Table 5) including cyanophage P60 and roseophage SIO1 [17,20]. An examination of the two genes *(nrdA* and *nrdB)* in P60 that contain homology to RNRs suggests that they represent a split RNR (as described earlier for DNAP): *nrdA* is similar to the 5′-end and *nrdB* is similar to the 3′-end of cyanobacterial class II RNRs (data not shown). When analyzed for the presence of a class II RNR diagnostic motif [64], all three marine T7-like phage putative RNRs were found to contain homology to this motif (seven of nine residues in SIO1, P-SSP7; eight of nine residues in P60; as compared to eight of nine residues in the marine cyanobacteria) (Figure S1). Furthermore, the putative RNRs are located in the genomes at the distal end of a region homologous to the nucleotide metabolism region in T7 [65]. It is plausible that T7-like phage infection in phosphorus-limited environments requires extra nucleotide-scavenging genes.

Both *Prochlorococcus* myoviruses contain the alpha and beta RNR subunits that are found in all known T4-like phages (see Table 4). The genes have closer sequence homology to those in T4-like phages than cyanobacterial hosts (Figure S2). Interestingly, our myoviruses also contain a noncyanobacterial *cobS* gene, which has never been found in phages. This gene encodes a protein that catalyzes the final step in cobalamin (vitamin B12) biosynthesis in bacteria [66,67], and cobalamin is an RNR cofactor during nucleotide metabolism in cyanobacteria [68]. Both physiological assays [69,70] and genomic evidence [4,34] indicate that *Prochlorococcus* synthesizes its own cobalamin. It is tempting to speculate that the phage *cobS* gene serves to boost cobalamin production in the host during infection, thus improving the activity of RNRs. However, these phage RNRs clearly contain the α2 and β2 subunits (typical of class I RNRs) and lack the class II motif described earlier. Thus, if the phage *cobS* does increase cobalamin production and if this production increase is important, then either the phage class I RNRs are cobalamin dependent (which is unprecedented) or cobalamin must be useful for some other process.

#### Carbon metabolism genes

In cyanobacteria, the pentose phosphate pathway oxidizes glucose to produce NADPH for biosynthetic reactions (oxidative branch) and ribulose-5-phosphate for nucleotides and amino acids (non-oxidative branch). This pathway (both branches) is particularly important in cyanobacteria for metabolizing the products of photosynthesis during dark metabolism [71]. Long ago, it was hypothesized that cyanophages utilize this pathway as a source of energy and carbon when the host is not photosynthesizing [72]. Interestingly, genomic sequencing has recently revealed that *Synechococcus* cyanophage S-RSM2 [16] and the *Prochlorococcus* cyanophages P-SSM2 and P-SSM4 [14] contain a transaldolase gene *(talC)*. In *Escherichia coli,* transaldolase is a key enzyme in the non-oxidative branch of the pentose phosphate pathway [73]. It has been suggested that the product of the phage *talC* gene may facilitate phage access to stored carbon pools during the dark period [16].

Recent work in E. coli has revealed two genes *(mipB/fsa* and *talC)* that are divergent from the bona fide transaldolases (*talA* and *talB*) [74], but encode a structurally similar enzyme [75]. Members of this new subfamily (MipB/TalC) of aldolases, which have a striking sequence similarity to each other, can have distinctly different functions, acting either as a transaldolase or fructose-6-phosphate aldolase, but not both [74]. All three of the genes previously reported as “transaldolase” genes in cyanophages [14,16], as well as an ORF in the podovirus P-SSP7, are most similar to these MipB/TalC aldolase genes (see Table 5; Figure S3). The translated cyanophage genes contain 26 (P-SSM2), 28 (P-SSP7 and S-RSM2), and 29 (P-SSM4) of 32 diagnostic (as designated by Thorell et al. [75]) amino acid residues (Figure S4). In the active site of this enzyme, as inferred from the crystal structure of E. coli fructose-6-phosphate aldolase, eight of 14 residues are not conserved between the MipB/TalC subfamily, varying depending on enzyme specificity (fructose-6-phosphate aldolase versus transaldolase) [75]. When aligned with MipB/TalC members of known substrate specificity, the cyanophage putative active site residues match all eight of those enzyme sequences with transaldolase activity (Figure S4). Thus, it appears that each of the four cyanophage *talC* genes encodes an enzyme with transaldolase activity. If functional, these genes are likely to be important for metabolizing carbon substrates—which is central to biosynthesis and energy production—during phage infection of cyanobacterial hosts.

#### Phosphate stress genes in the myoviruses

Phosphorus is a scarce resource in the oligotrophic oceans [76,77]. It is often growth limiting for cyanobacteria [78] and is required in significant amounts for phage replication. Thus it is perhaps not surprising that the phosphate-inducible *phoH* gene, which has been found in two marine phage genomes [20,21], is also found in both *Prochlorococcus* myoviruses (see Table 5; see Figures 3 and 4). Although the *phoH* gene is found widely distributed among both eubacteria and archaea [79], including all cyanobacteria, and is known to be induced under phosphate stress in E. coli [80], its function has not been experimentally determined. Bioinformatic analyses suggest that these *phoH* genes are part of a multi-gene family with divergent functions from phospholipid metabolism and RNA modification (COG1702 *phoH* genes) to fatty acid beta-oxidation (COG1875 *phoH* genes) [79].

Both P-SSM2 and P-SSM4 also contain a phosphate-inducible *pstS* gene—which is also widespread among the archaea and eubacteria, including all known cyanobacteria—that has not been reported in phages. In bacteria, the *pstS* gene encodes a periplasmic phosphate-binding protein involved in phosphate uptake [81]. If expressed by the phage, it might serve to enhance phosphorus acquisition during infection of phosphate-stressed cells.

#### LPS biosynthesis genes in P-SSM2

The myovirus P-SSM2 contains 24 LPS genes that form two major clusters in the genome (see Figure 3). Reports of phage-encoded LPS genes have previously been limited to temperate phages [82]. Such temperate phage LPS genes are thought to be used during infection and establishment of the prophage state to alter the cell-surface composition of the host, preventing other phages from attaching to the host cell. Although T4-like phages are commonly thought of as lytic phages, the lytic process can be stalled upon infection (sometimes termed “pseudolysogeny”) during suboptimal host growth [83]. If this phenomenon occurs in marine phages, as has been suggested [22,84,85], then a phage-encoded LPS gene cluster, even in a lytic phage, might maintain a similar functional role.

#### Signature genes for oceanic cyanophages?

Although data are too limited to be conclusive (Table 6), some of the host genes that appear common in oceanic cyanophages may ultimately represent signature genes for these phages. For example, the genomes of all three cyanophages presented here and five partial genomes (<5 kb) of *Synechococcus* cyanomyophages presented by Millard et al. [16] all contain a *psbA* gene. Further, all three cyanophages presented here contain at least one *hli* and a *talC* gene, and both myoviruses presented here are unique among the phages in that they contain *pstS* and *cobS* (Table 6). As more phages are sequenced, will we find that these genes are specifically characteristic of oceanic cyanophages? If true, this would provide us with a powerful tool for studying these phages in the wild because quantitative PCR could be used to differentiate between cyanophages and other phages in environmental samples.

**Table 6:**
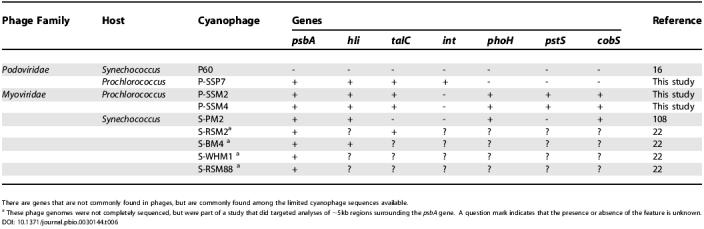
Signature Cyanophage Genes?

There are genes that are not commonly found in phages, but are commonly found among the limited cyanophage sequences available

^a^ These phage genomes were not completely sequenced, but were part of a study that did targeted analyses of ∼5kb regions surrounding the *psbA* gene. A question mark indicates that the presence or absence of the feature is unknown

### Hypothesized Transient Genes

There are genes of interest, found in only one of the myoviruses, that we hypothesize are not functional, but rather were obtained by cyanomyophages through packaging random DNA, probably by illegitimate recombination [86,87] with DNA from a common phage genome pool [88].

#### Trytophan halogenase

P-SSM2 contains a gene *(prnA)* that is known to exist in only nine species of bacteria, in which it encodes a tryptophan halogenase that catalyzes the NADH-consuming first step of four that are involved in converting tryptophan to the antibiotic pyrrolnitrin [89,90,91]. Although this gene is full length (Figure S5), *prnA* is part of a unique metabolic pathway missing in most bacteria, including cyanobacteria.

#### Archaeal and eukaryotic genes.

The other myovirus, P-SSM4, contains three grouped genes with homology only to eukaryotic prion-like proteins (orf32), an archaeal protease (orf35), and a hypothetical protein from a eukaryotic slime mold (orf36) (see Figure 4). Other eukaryotic and prion-like genes have been predicted in the genomes of mycobacteriophages that infect actinobacterial hosts [92], although they have no similarity to those found in P-SSM4.

#### Hemagglutinin neuraminidase

P-SSM4 contains a possible hemagglutinin neuraminidase (HN), which has only been observed in single-stranded RNA (ssRNA) viruses and *Prochlorococcus* MED4 (orf1400). In ssRNA viruses, HN cleaves sialic acid from glycolipids on the host cell surface, which enables these viruses to attach. Protein alignments show, however, that both the MED4 and P-SSM4 HN genes are only partial genes—they are missing the N- and C-termini (approximately 200 amino acids)—relative to other ssRNA HNs (Figure S6). It is noteworthy that the HN gene occurs nowhere else in the prokaryotic world except for MED4. Could this gene have been obtained by P-SSP7 through the phage genome pool (sensu Hendrix et al. [88]), then transferred to MED4? This postulate is buttressed by the observation that the HN gene in MED4 is found next to three *hli* genes (which encode high-light-inducible proteins)—genes which we have argued earlier are susceptible to horizontal gene transfer in this phage–host system [14].

### Ecological and Evolutionary Implications of Phages Carrying Host Genes

*Prochlorococcus* cells are slow-growing (doubling times range from 1 to 10 d), oxygenic phototrophs that thrive in nutrient-poor, aerobic surface waters [1]—conditions that are fundamentally different from those of most of the host cells of the phages sequenced to date. Thus, oceanic cyanophages are subject to substantially different selective pressures than most other sequenced phages in the database. The presence in these phages of host genes that are likely involved in the maintenance of photosynthesis, response to phosphate stress, and mobilization of carbon stores during infection may be interpreted as evidence of such unique pressures (see Table 5).

If phage genomes interact as “local neighborhoods” (sensu Hendrix et al. [88]) within a “global phage metagenome” (sensu Rohwer [93]), one would expect to find biologically cohesive units akin to species, defined by local gene transfers as proposed for “microbial species” [94]. Such cohesive units would be characterized by core genes that determine a general phage infection lifestyle (e.g., T4-like or T7-like), as well as host-specific genes within phages that infect similar hosts. Indeed, 26 and 75 such core genes exist among the T7-like and T4-like phages, respectively (see Tables 3 and 4), and host-specific genes abound among these cyanophages (see Figures 1C, 5A, and 5B). That these core genes represent mostly morphological and DNA replication genes suggests a T7-like or T4-like lifestyle that would involve a specific means of delivering DNA from host to host (in a tailed, capsid structure) as well as converting the host into a phage factory. Based upon the presence of many such core genes in our *Prochlorococcus* phages, one would predict they would behave as T7-like (P-SSP7; although probably with the ability to integrate into its host) and T4-like phages (P-SSM2 and P-SSM4) during cyanobacterial infection.

Beyond these core genes, our *Prochlorococcus* phages contain many “nonphage” genes that are of greatest sequence similarity to cyanobacterial genes (see Figures 1C, 5A, and 5B). We speculate that the acquisition and use of some host genes by phages plays an important role in phage ecology, even *shaping* the evolution of the phage host range. The initial host range alterations are likely to occur by phage tail fiber switching [47], but beyond that, these co-opted host genes could either shift or expand the phage's host range depending upon whether they affect fitness of the phage in the original hosts. Understanding this dynamic fitness landscape will require modeling efforts directed by a thorough knowledge of the mechanisms and relative rates for this complex genetic shuffling—factors that likely underpin the complexity of phage–host interactions in the environment.

## Materials and Methods

### 

#### Electron microscopy

*Prochlorococcus* phages were concentrated using ultracentrifugation. Concentrates were prepared for microscopy by spotting phage lysates onto freshly glow-discharged carbon/formvar–coated copper grids. Grids were negatively stained with 1% uranyl acetate, dried, and viewed in a JEOL (Peabody, Massachusetts, United States) 1200 EXII transmission electron microscope operated at 80 kV.

#### Preparation of cyanophages for genome sequencing

Three *Prochlorococcus* phages were chosen for sequencing based upon their host ranges, which were restricted to *Prochlorococcus* hosts (see Introduction).

Phages were prepared for genomic sequencing as previously described [14,95]. Briefly, phage particles were concentrated from phage lysates using polyethylene glycol. Concentrated DNA-containing phage particles were purified from other material in phage lysates using a density cesium chloride gradient. Purified phage particles were broken open (SDS/proteinase K), and DNA was extracted (phenol:chloroform) and precipitated (ethanol) yielding small amounts of DNA (<1 μg). A custom 1- to 2-kb insert linker-amplified shotgun library was constructed by Lucigen (Middletown, Wisconsin, United States) as described previously [95]. Additional larger insert (3–8 kb) clone libraries were constructed from genomic DNA by the Department of Energy (Joint Genome Institute, Walnut Creek, California, United States) using a similar protocol to provide larger scaffolds during assembly. Inserts were sequenced by the Department of Energy Joint Genome Institute from all of these clone libraries and used for initial assembly of these phage genomes. The Stanford Human Genome Center Finishing Group (Palo Alto, California, United States) closed the genomes using primer walking.

#### Gene identification and characterization

Protein coding genes were predicted using GeneMark [96] and manual curation. Translated ORFs were compared to known proteins in the nonredundant GenBank database (http://www.ncbi.nlm.nih.gov/BLAST/) and in the KEGG database (http://www.genome.ad.jp/kegg/kegg2.html) using the BLASTp program (ftp://ftp.ncbi.nih.gov/blast). Translated ORFs were also analyzed for signal sequences and transmembrane regions using the Web-based software SignalP and TMHMM, respectively (available at the CBS prediction servers; http://www.cbs.dtu.dk/services/). Where BLASTp e-values were high (>0.001) or no sequence similarity was observed, ORF annotation was aided by the use of PSI-BLAST, gene size, domain conservation, and/or synteny (gene order), the last as suggested for highly divergent genes encountered during phage genome annotation [97]. Identification of tRNA genes was done using tRNAscan-SE [98].

#### Taxonomy of best hits

For global genome comparison, we used BLASTp (e-values < 0.001) or manual annotation to classify to which group of organisms or phages each predicted coding sequence was most similar. In most cases this was obvious. However, approximately 2% of the coding sequences were less obvious, so we established an operational definition of “most similar” as the query sequence having e-values within four orders of magnitude of the top cluster of organismal types. For example, if a query sequence was similar to noncyanobacterial sequences with e-values of 10^–29^ to 10^–25^ and to cyanobacterial sequences with e-values of 10^–20^ or greater, then, despite sequence similarity to cyanobacterial sequences, the query would be considered noncyanobacterial.

#### Tail fiber gene identification

Tail fiber genes were identified by generating alignments (stand-alone Basic Local Alignment Search Tool, BLAST [99], 2.2.8 release) of conceptually translated, computationally identified ORFs from the P-SSM2 and P-SSM4 genomes against a database consisting of 33,270 sequences encompassing all known phage sequences obtained from the NCBI NR database in April 2004. Only ORFs whose alignments to known tail fiber genes were longer than 100 residues and had e-values less than 0.001 were designated as tail-fiber-like. Sequences close to this cutoff were re-aligned using the bl2seq command of BLAST, which computes e-values independently of database size. Tail-fiber-like paralogs were identified by individually aligning the set of tail-fiber-like ORFs with all other ORFs in the genomes. All ORFs with alignments greater than 100 residues and e-values less than 0.001, were designated as tail fiber paralogs. All BLAST searches and alignments were performed with the low-complexity sequence filter and default parameters. Amino acid sequence repeats were identified by self-alignment matrices using the program Dotter [100].

#### Sequence manipulation and phylogenetic analyses

Alignments were generated using Clustal X [101] and edited manually as necessary. PAUP V4.0b10 [102] was used for the construction of distance and maximum parsimony trees. Amino acid distance trees were inferred using minimum evolution as the objective function, and mean distances. Heuristic searches were performed with 100 random addition sequence replicates and the tree bisection and reconnection branch-swapping algorithm. Starting trees were obtained by stepwise addition of sequences. Bootstrap analyses of 1,000 resamplings were carried out. Maximum likelihood trees were constructed using TREE-PUZZLE 5.0 [103]. Evolutionary distances were calculated using the JTT model of substitution assuming a gamma-distributed model of rate heterogeneities with 16 gamma-rate categories empirically estimated from the data. Quartet puzzling support was estimated from 10,000 replicates.

## Supporting Information

Figure S1 Class II RNR Motif Compared Against Cyanobacterial and Non-T4-Like Phage RNRs A question mark indicates this sequence data is not known; a period indicates identical residue to the reference sequence; and a dash indicates a gap in the alignment. *Anab, Anabaena; Pro, Prochlorococcus; Syn, Synechococcus*; *Syncy*, *Synechocystis*.
(10 KB PDF).

Figure S2 Distance Tree of RNR Family Proteins, Including Phage Sequences from P-SSM2, P-SSM4, and P-SSP7 Sequences from P-SSM2, P-SSM4, and P-SSP7 are shown in bold. Trees were generated from 900 amino acids. Bootstrap values for distance and maximum parsimony analyses and quartet puzzling values for maximum likelihood analysis, greater than 50%, are shown at the nodes (distance/maximum likelihood/maximum parsimony). Trees were unrooted; abbreviations as in Figure S1.
(14 KB PDF).

Figure S3 Distance Tree of Tal Proteins, Including Phage Sequences from P-SSM2, P-SSM4, and P-SSP7 Sequences from P-SSM2, P-SSM4, and P-SSP7 are shown in bold. Trees were generated from 566 amino acids. Bootstrap values for distance and maximum parsimony analyses and quartet puzzling values for maximum likelihood analysis, greater than 50%, are shown at the nodes (distance/maximum likelihood/maximum parsimony). Trees were unrooted; abbreviations as in Figure S1.
(14 KB PDF).

Figure S4 Alignment of TalC Subfamily Aldolases, Including Phage Sequences from P-SSM2, P-SSM4, P-SSP7, and S-RSM2 The 32 amino acid residues suggested to be diagnostic by Thorell et al. [75] are labeled with an asterisk and shaded where identical to bona fide TalC proteins, whereas the active site residues are labeled with an “at” symbol. Note the active site residues in the cyanophage TalC sequences exclusively match those from enzymes known to have transaldolase activity rather than fructose-6 phosphate aldolase activity.
(14 KB PDF).

Figure S5 Alignment of Tryptophan Halogenase Amino Acid Sequences Deduced from Phage and Cellular Encoded *prnA* Gene Sequences Note the phage gene appears full-length relative to the other cellular genes. *Bdellovibrio, Bdellovibrio bacteriovorus; Bordtella, Bordetella pertussis; Burkpyrro, Burkholderia pyrrocinia; Caulobacter, Caulobacter crescentus; Myxfulvus, Myxococcus fulvus; Pschloro, Pseudomonas chlororaphis; Pseud_fl, Pseudomonas fluorescens; Shewanella, Shewanella oneidensis* MR-1; *Xanaxon, Xanthomonas axonopodis; Xancamp, Xanthomonas campestris.*
(35 KB PDF).

Figure S6 Alignment of HN Amino Acid Sequences Deduced from Phage and ssRNA Viral Gene Sequences Note the *Prochlorococcus* phage and host gene appears to contain only the central region of the gene relative to the other ssRNA viral genes.APMV6, avian paramyxovirus 6; BPIV3, bovine parainfluenza virus 3; Gparamyxovirus, goose paramyxovirus; HPIV1,2,3, human parainfluenza virus 1,2,3; ProMED4, *Prochlorococcus* MED4.
(36 KB PDF).

### Accession Numbers

The GenBank (http://www.ncbi.nlm.nih.gov/Genbank/) accession numbers for the genomes discussed in this paper are MED4 (BX548174), P-SSM2 (AY939844), P-SSM4 (AY940168), and P-SSP7 (AY939843).

## Abbreviations

DNAP: deoxyribonucleic acid polymerase
HL: high-light-adapted
HN: hemagglutinin neuraminidase
LL: low-light-adapted
LPS: lipopolysaccharide
ORF: open reading frame
PSII: photosystem II
RNAP: ribonucleic acid polymerase
RNR: ribonucleotide reductase
ssRNA: single-stranded ribonucleic acid
