# Supplementary material for: Three Prochlorococcus Cyanophage Genomes: Signature Features and Ecological Interpretations
Source: PLoS Biol. 2005 Apr 19;3(5):e144. doi: 10.1371/journal.pbio.0030144 (PMC1079782; doi:10.1371/journal.pbio.0030144)
Supplement: Supplementary file 1 [file pbio.0030144.sg001.pdf]

Supplementary Figure 1: Comparison of Cyanobacterial and non-T4-like phages against class II RNR motif.

**Class II RNR MOTIF**	**T/S**	**N**	**P**	**C**	**G/S**	**E**	**X**	**X**	**X**	**X**	**X**	**X**	**X**	**X**	**C**	**N/L**	**L**
Thermosynechococcus	L	.	.	.	G	.	I	I	-	G	A	D	F	H	.	N	.
ProMED4	L	.	.	.	G	.	I	L	-	G	N	D	F	H	.	N	.
ProMIT9313	L	.	.	.	G	.	I	L	-	G	A	D	F	H	.	N	.
ProSS120	L	.	.	.	G	.	I	L	-	G	A	D	F	H	.	N	.
SynWH8102	L	.	.	.	G	.	I	L	-	G	A	D	F	H	.	N	.
Cyanophage P60_nrdB	L	.	.	.	G	.	I	L	-	G	K	D	F	H	.	N	.
Mycobacteriophage D29	T	.	.	.	G	.	I	T	L	E	P	W	E	P	.	N	.
Roseophage SIO1	V	.	.	.	A	.	I	L	L	G	N	K	S	F	.	N	.
Cyanophage P-SSP7	S	.	V	.	L	.	V	Y	L	P	S	R	G	T	.	L	.
SS (GI:44265515)	G	.	V	.	L	.	V	Y	L	P	S	R	G	T	.	L	.
SS (GI:44494788)	G	.	V	.	L	.	V	Y	L	P	S	R	G	T	.	L	.
SS (GI:43424236)	?	?	?	.	L	.	V	Y	L	P	S	R	G	T	.	L	.
