# Supplementary material for: Three Prochlorococcus Cyanophage Genomes: Signature Features and Ecological Interpretations
Source: PLoS Biol. 2005 Apr 19;3(5):e144. doi: 10.1371/journal.pbio.0030144 (PMC1079782; doi:10.1371/journal.pbio.0030144)

Supplementary Figure 2: Distance tree of ribonucleotide reductase proteins

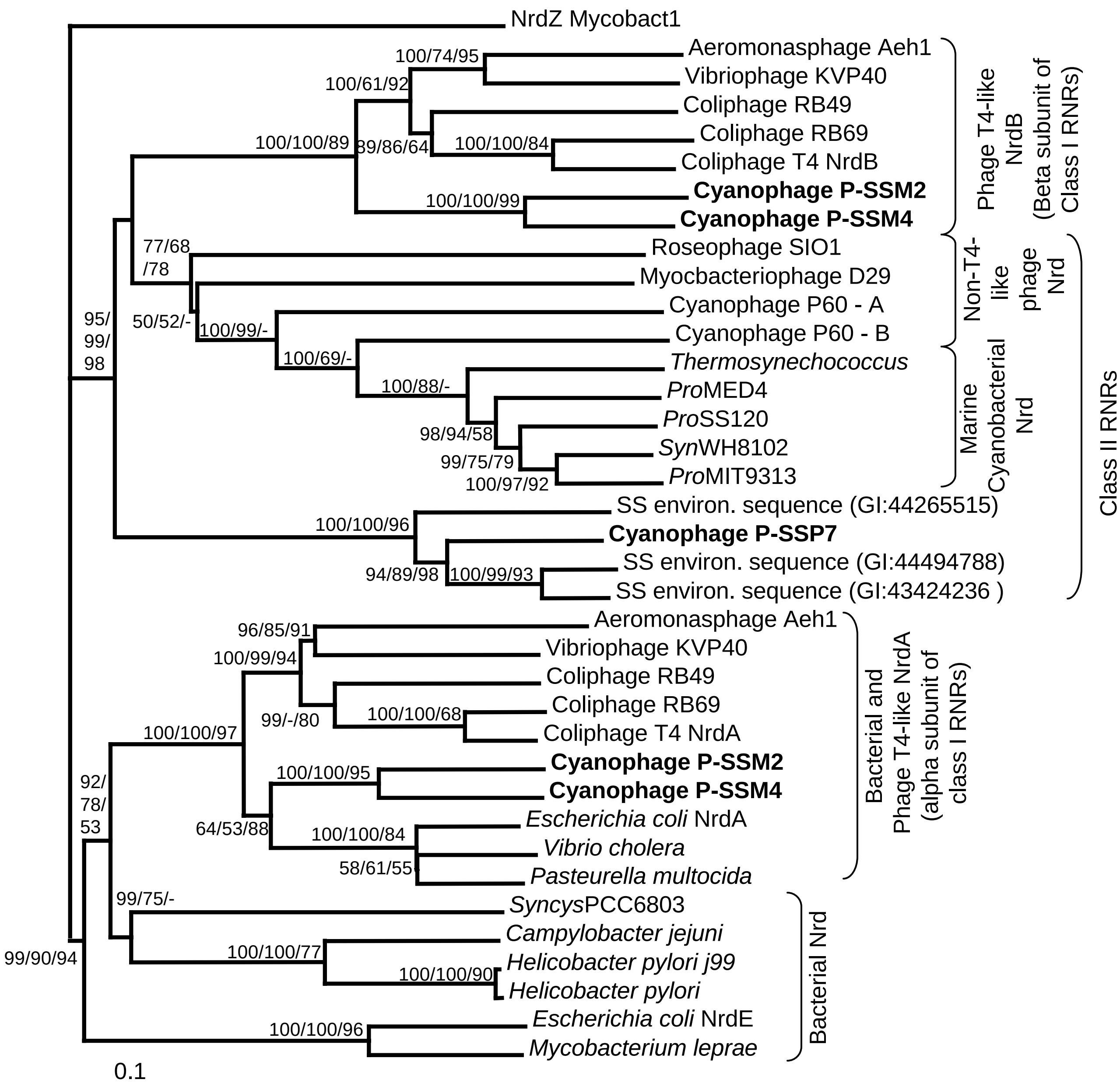

Supplement: Supplementary file 2 [file pbio.0030144.sg002.pdf]
