# Supplementary material for: Three Prochlorococcus Cyanophage Genomes: Signature Features and Ecological Interpretations
Source: PLoS Biol. 2005 Apr 19;3(5):e144. doi: 10.1371/journal.pbio.0030144 (PMC1079782; doi:10.1371/journal.pbio.0030144)
Supplement: Supplementary file 4 [file pbio.0030144.sg004.pdf]

Supplementary Figure 4: Alignment of TalC Subfamily aldolases, including phage sequences from P-SSM2, P-SSM4, P-SSP7 and P-RSM2

```
                            @                   @ @                             @ @
S_RSM2             1   MKIFLDTADTNVIKEYFETGLVDGVTTNPTLIMKSG-RNPEEVYQEIKDIG--VEDISMEVMGTAAEMYH 67
P_SSM2             1   MKIFLDTADTDAIKQHYDTGIIDGITTNPTLIRKSG-RDPEEVYQELIDYG--INDISMEVVGDYGTMFE 67
P_SSM4             1   MKLFLDCSDAEFIRDAYSTGLIDGVTTNPSLMLKAG-KDPREVLKEISDIFPFHASVSAEVVGDSVEEML 69
P_SSP7             1   MKIFLDSAITTDIQDRLATEIIDGVTTNPTLIKKSN-EDPDVVYKELYDMR--VKDLSIEVRGETAQELC 67
M.jannaschii       1   MKFFLDTANVEEIKKYAELGLVDGVTTNPTLVAKEG-RDFYEVVKEICEIV--EGPVSAEVISTDAEGMV 67
T.maritima         1   MKIFLDTANLEEIKKGVEWGIVDGVTTNPTLISKEG-AEFKQRVKEICDLV--KGPVSAEVVSLDYEGMV 67
B.subtilis         1   MLFFVDTANIDEIREANELGILAGVTTNPSLVAKEANVSFHDRLREITDVV--KGSVSAEVISLKAEEMI 68
E.coli_mipB        1   MELYLDTSDVVAVKALSRIFPLAGVTTNPSIIAAGK-KPLDVVLPQLHEAMGGQGRLFAQVMATTAEGMV 69
E.coli_talC        1   MELYLDTANVAEVERLARIFPIAGVTTNPSIIAASK-ESIWEVLPRLQKAIGDEGILFAQTMSRDAQGMV 69
Diagnostic residues    *    *                 *** **                         *  * *        *

                                       @                     @ @@ @                @ @  @
S_RSM2             68  EGRRLH-LKFGDVATIKVPCTRDGLSVCKQLSDEGIKVNVTLIFCAAQAVLAAKAGATYVSPFVGRLDDQ 136
P_SSM2             68  EGTRLS-RKFGKACTVKVPCTPDGLKVCRELSRDLVNVNVTLIFSAAQAILAAKSGAKYVSPFVGRVDDN 136
P_SSM4             70  EMADDY-IEIGPNITIKVPLTPEGLKVCKDLSTDDVAVNVTLCFSTAQAILAAKAGATYVSPFVGRVNDQ 138
P_SSP7             68  ANGILYGRKYGEVATIKLPCTVEGLKACKKLSILGHKTNMTLVFSVSQAILCAHAGATYISPFVGRLDQI 137
M.jannaschii       68  KEAREL-AKLADNIVIKIPMTKDGMKAVKILSAEGIKTNVTLVFSPLQALVAAKAGATYVSPFVGRLDDI 136
T.maritima         68  REAREL-AQISEYVVIKIPMTPDGIKAVKTLSAEGIKTNVTLVFSPAQAILAAKAGATYVSPFVGRMDDL 136
B.subtilis         69  EEGKEL-AKIAPNITVKIPMTSDGLKAVRALTGLGIKTNVTLIFNANQALLAARAGATYVSPFLGRLDDI 137
E.coli_mipB        70  NDALKL-RSIIADIVVKVPVTAEGLAAIKMLKAEGIPTLGTAVYGAAQGLLSALAGAEYVAPYVNRIDAQ 138
E.coli_talC        70  EEAKRL-RDAIPGIVVKIPVTSEGLAAIKILKKEGITTLGTAVYSAAQGLLAALAGAKYVAPYVNRVDAQ 138
Diagnostic residues                    * *    *  *             *      *  *   *** *  *   *

                                                     @
S_RSM2             137 SVAGLEVVRSISELYRIHGIR-TQVLSASIRSVQRAIRSWYNGAEICTMPPKVFDQMYDHILTDKGMEIF 205
P_SSM2             137 SFVGMDLIEQISDIYTIQNVHKTEILSASIRDVKSVSDSFASGAHVVTMPPTVFEKMYNHVLTDKGLYLF 206
P_SSM4             139 SFDGIKLIEEISDVYATHKQK-TQVLAASIRDVYQVASCFRVGADICTIPSKIFSGMYKHILTDQGIAKF 207
P_SSP7             138 GEDGIQLIQDIAKVFCIHNIE-TQILAASIRSPKQAEDAYKAGAHICTLPVKVFDLMFRHHLTDEGLKQF 206
M.jannaschii       137 GHVGMKLIEDVVKIYKNYDIK-TEVIVASVRHPWHVLEAAKIGADIATMPPAVMDKLFNHPLTDIGLERF 205
T.maritima         137 SNDGMRMLGEIVEIYNNYGFE-TEIIAASIRHPMHVVEAALMGVDIVTMPFAVLEKLFKHPMTDLGIERF 205
B.subtilis         138 GHNGLDLISEVKQIFDIHGLD-TQIIAASIRHPQHVTEAALRGAHIGTMPLKVIHALTKHPLTGKGIEQF 206
E.coli_mipB        139 GGSGIQTVTDLHQLLKMHAPQ-AKVLAASFKTPRQALDCLLAGCESITLPLDVAQQMISYPAVDAAVAKF 207
E.coli_talC        139 GGDGIRTVQELQTLLEMHAPE-SMVLAASFKTPRQALDCLLAGCESITLPLDVAQQMLNTPAVESAIEKF 207
Diagnostic residues                               **              *    *            *        *

S_RSM2             206 ENDWKGVQK---- 214
P_SSM2             207 DMDWAQVKR---- 215
P_SSM4             208 DEDWTKLVGG--- 217
P_SSP7             207 ALDFGRNV----- 214
M.jannaschii       206 LKDWDEYLKSRK- 217
T.maritima         206 MEDWKKYLENLKK 218
B.subtilis         207 LADWNK------- 212
E.coli_mipB        208 EQDWQGAFGRTSI 220
E.coli_talC        208 EHDWNAAFGTTHL 220
Diagnostic residues      **
```
