# Supplementary material for: Three Prochlorococcus Cyanophage Genomes: Signature Features and Ecological Interpretations
Source: PLoS Biol. 2005 Apr 19;3(5):e144. doi: 10.1371/journal.pbio.0030144 (PMC1079782; doi:10.1371/journal.pbio.0030144)
Supplement: Supplementary file 5 [file pbio.0030144.sg005.pdf]

Supplementary Figure 5: PrnA protein alignments

```
                      10        20        30        40        50        60        70        80        90       100
              ....|....|....|....|....|....|....|....|....|....|....|....|....|....|....|....|....|....|....|....|
Xanaxon       ---------------------MIPAPLRNIVIVGGGTAGWMAAAAFARVLGP--TFKVQLIESEQLGTVGVGEATVP-HIKAFNNLLGIDEAEFVRQTQG
Xancamp       ---------------------MIPAPLRNIVIVGGGTAGWMAAAAFARVLGP--SFNVQLIESEQLGTIGVGEATVP-HIKAFNNLLGIDEAEFVRQTQG
Caulobacter   --------------------MSQDHRLRRIVIVGGGTSGWMTAAALGRFLKDG-YTQVTLVESEAIGTVGVGESTIP-QINIFNRMLGLDENDFVRKTKA
Pseud_fl      ----------------------MNKPIKNIVIVGGGTAGWMAASYLVRALQQ--QVNITLIESAAIPRIGVGEATIPSLQKVFFDFLGIPEREWMPQVNG
Burkpyrro     ----------------------MSNPIKNIVIVGGGTAGWMAASYLVRALQQ--QTNITLIESAAIPRIGVGEATIPSLQKVFFDFLGIPEREWMPQVNG
Pschloro      ----------------------MNKPIKNIVIVGGGTAGWMAASYLVRALQQ--QANITLIESEAIPRIGVGEATIPSLQKVFFDFLGIPEREWMPQVNG
Myxfulvus     ---------------MDQSSVENDNRVRKIIIVGGGSSGWMAAAYLSKALNF--NADITLIESPTIPRIGVGEATIPTIKEELFDFLEIPEEEWMSECKA
Shewanella    ------------------------MTIRKIAIIGGGTSGWLAANHLGRALKGRSGLSISVIESPDIPIIGVGEGTVPSIRKSLQSFG-ISESEFIRTCDV
Bdellovibrio  MSTLSEHIDNAFDLSYLNFPKCPPTDIKTIGILGGGTAGYLAALALKKYHPE---VEVTVIESSKIPVIGVGESTTTEIVPFLHRGLEIDPAEFFREVEP
P-SSM2        ------------------------MSDSDFIVVGGGNAGCISALLLQCSFPN---KKVSIIKSENIGTVGVGE-SSTEHFAQFCNTCGISILNVLLATKA
Bordetella    ----------------------------------------------------------------------------------------------------
Consensus

                     110       120       130       140       150       160       170       180       190       200
              ....|....|....|....|....|....|....|....|....|....|....|....|....|....|....|....|....|....|....|....|
Xanaxon       SFKLGIEFVDWQR-----PGSTYMHGFGTQIGHPLGLLPFHQYWIKQHA-RGKAQPLGAYTLNTVAAARGKFMTSAGDVPANSLLANIAYAYHFDATQYA
Xancamp       SFKLGIEFIDWQR-----PGTSYIHGFGTQIGHPLGLLPFHQYWIKQQR-RGKAQPLGAYTLNTVAATRGKFMTSAGDVPANSPLANIAYAYHFDASQYA
Caulobacter   TFKLGIDFVDWQR-----IGHTYHHPFG-PYGVDMEGVSFHAYWLKLRA-MGLDDDLGAYSLQTVGARQSKFMRPNG--QANSPLGQIAYAFQFDAGLYA
Pseud_fl      AFKAAIKFVNWRKSPDPSREDYFYHLFG--SVPNCDGVPLTHYWLRKRE-QGFQQPMAYACYPQPGALDGKLA-----PCLADGTRQMSHAWHFDAHLVA
Burkpyrro     AFKAAIKFVNWRKSPDRSRDDHFYHLFG--SVPNCDGVPLTHYWLRKRE-QGFQQPMEYACYPQPGALDGKLA-----PCLSDGTRQMSHAWHFDAHLVA
Pschloro      AFKAAIKFVNWRKSPGSSRDDHFYHLFG--NVPNCDGVPLTHYWLRKRE-QGFQQPMEYACYPQAGALDGKLA-----PCLPDGTRQMSHAWHFDAHLVA
Myxfulvus     TYKLGIRFQNWKK-PASQGGDHYYHNFG--EMPQVKGVPLSHLWMYKHQLQGFAEPMAYSCYPTSPICDAQKS-----PRYMDGTKAVHYAYHFDALLVA
Shewanella    TFKQSIKFVNWLDKTRHGKHNFYHHLFD---IINLHQGDAVSAWLAEQS-GHFADFVSTQHLACEVAKAPKLIT------TPEYAGVLGYAYHLNAAKFA
Bdellovibrio  TLKFGIRFDWGCP-------GDYKFNFS------FFAGHQYESYYYENDIKNSNWPSVLMNENKIPVIRNKNG------TLTSFLASIPFSYHIDNKNLI
P-SSM2        TFKGGVYFENWSD-------EDFMHVVN----LDNVNQPIGHFFPYLQRVVAYNKPNREMNIYGAWDRKVPLK------FFNDMNNSPTNQFHFDTLALN
Bordetella    ----------------------------------------------------------------------------------------------------
Consensus

                     210       220       230       240       250       260       270       280       290       300
              ....|....|....|....|....|....|....|....|....|....|....|....|....|....|....|....|....|....|....|....|
Xanaxon       HFLRRYSQQRGVTRIEGLVEQVQLHPESGHVQSLQLASGQVVHGDLFIDCSGFGGLLIEQALHTGYHDFTHWLPCDRALAVPCEQVSP---PAPYTRSTA
Xancamp       RFLRGYAEQRGVTRLEGLIEQVQLDAHTGHVQSVQLASGQVVSGDLFIDCSGFRGLLIEEALHTGYHDFTHWLPCDRALAVPCEKVGP---PTPYTRSTA
Caulobacter   KFLRDYSEARGVTRQEGKIASVQQDGQSGHVTSVTLESGQVIEGDLFIDCSGFRGLLIEQTLKTGYEDWSQWLLNDRAVAMPCTLGGS---MAPVTRATA
Pseud_fl      DFLKRWAVERGVNRVVDEVVEVQLN-DRGYISTLLTKEGRTLEADLFIDCSGMRGLLINQALKEPFIDMSDYLLCDSAVASAVPNDDAREGVEPYTSAIA
Burkpyrro     DFLKRWAVERGVKRVVDEVVEVRLN-DRGYISSLSTKEGRTLEADLFIDCSGMRGLLINQALKEPFIDMSDYLLCDSAVASAVPNADARVGVEPYTSAIA
Pschloro      DFLKRWAVERGVKRVVDEVVDVHLN-ERGYISSLFTKEGRTLEADLFIDCSGMRGLLINQALKEPFIDMSDYLLCDSAVASAVPNDDAREGVEPYTSAIA
Myxfulvus     NFLKKWSIERGLTFISDDIVDTQLD-DGGSIKHLRGASGRSYAADLYIDCTGFAGLLIEQALGEPKVTFHDSLLTDRAVAINIPSDPATEGIRPYTTASA
Shewanella    KLLAKNAIEKFNVEHILTTVHDVCLGVDGAIESLQTEQGN-LSFDFYIDCSGFESILLAKALKVPFISKAQQLFIDTALVAQIP-TQPADVIPPYTKATA
Bdellovibrio  RFLNKEVKSRGIDILDAEVHEVKLD-ERGYVASLESKEGHSLSYDLYIDCSGFRSKILGQALKTEFTPFTDTLVTDRALTFDMPNKDV---VDCYTSVIT
P-SSM2        KFLIDKCLERNINIIEDELMGAIVHPESGDIVSVNG-ETDKYSADFFIDCSGFSRLLLEKTLGVKWKSYSEYLPLNSAISFATDEMEEYN---MYTKSTA
Bordetella    -MFSLQEQTAILAHLNVRTERHGDEPAGGADLKISFTTGNGILSEFHPRLR--HGLYKAEEAPNQAEMAVGEALTERIYADLIDRVRLKHDLKGAKVLIG
Consensus      ::                         *    :          ::.            :               :                   .

                     310       320       330       340       350       360       370       380       390       400
              ....|....|....|....|....|....|....|....|....|....|....|....|....|....|....|....|....|....|....|....|
Xanaxon       RAAGWQWRIPLQHRTGNGYVYCSAHISDDEAAATLLANLD-GKPLADPRPLRFTTGRRKQCWNRNVVALGLASGFLEPLESTSIHLIQSGISKLLELFPR
Xancamp       RAAGWQWRIPLQHRTGNGYVYSSAHVSDDEAAATLLANLD-GTPLADPRPLRFTTGRRKQFWNRNVVALGLASGFLEPLESTSIHLIQSGISKLLELFPR
Caulobacter   RPHGWQWRIPLQHRLGNGYAYSSRHVSDDEALADLLSQLD-GEPLADPNLIRFTPGRRKKSWNGNVVAIGLSAGFMEPLESQSIYLIQVGISRLLAHFPD
Pseud_fl      MNSGWTWKIPMLGRFGSGYVFSTKFTSRDQATADFLKLWG-LSDNQQLNQINFRVGRNKRAWVNNCVSIGLSSCFLEPLESTGIYFIYAALYQLVKHFPD
Burkpyrro     MNSGWTWKIPMLGRFGSGYVFSSKFTSRDQATADFLNLWG-LSDNQPLNQIKFRVGRNGRAWVNNCVAIGLSSCFLEPLESTGIYFIYAALYQLVKHFPD
Pschloro      MNSGWTWKIPMLGRFGSGYVFSSKFTSRDQATADFLNLWG-LSDNQPLNQIKFRVGRNKRAWVNNCVSIGLSSCFLEPLESTGIYFIYAALYQLVKHFPD
Myxfulvus     FSSGWTWEIPLYGRAGNGYVYSSAFQTPEDAEREVRAWFGKKADALDVRHIKFVSGRRRRSWVKNCVSIGLASSFLEPLESTGLYFVYAALYQLVACFPS
Shewanella    HQAGWIWDIALTQRRGTGFVYSSAHMNQAEAERKFDHYLGGKLADIPHRKIPMTVGYRQQFWVKNCVALGLAQGFLEPIEATSILLTDFSARFLAERFPA
Bdellovibrio  MNNGWCWKIPMRTEDHYGYVFSSKFCDEETALAEARARFG---HFEKYKMVEFRSGRHTQAWVKNVFSLGNSYGFIEPLESTAIQTAVHSINVLCRLMPK
P-SSM2        RDYGWSWQIPTQGRTGNGYVFSEKFINETQAHEEMEKSYG--HEIDVAKTFKFDPGRLEKAWCKNCYAVGLSQSFVEPLEATSIGSVIQQMVAFIHYYPS
Bordetella    FGLGGSSDIQMDPVDVDGFQAELHEGGSVTLTFRVKCHPT----GEQVKKLHEVLGNEITISVTPAIEKQGALGLNLEPEAA------------------
Consensus        *    *        *:                             . .    *               :  :    *:

                     410       420       430       440       450       460       470       480       490       500
              ....|....|....|....|....|....|....|....|....|....|....|....|....|....|....|....|....|....|....|....|
Xanaxon       -EGISPVLVQRYNDRLAFEFDRIRDFLLLHYHATERDDSAFWRHCR-HMPITPELQATLDLFRD--------------SGRFYRNAEEMFAEISWVQVLV
Xancamp       -ESISPVLVQRYNDRLAFEFDRIRDFLVLHYHATERDNSAFWRHCR-TQPITPELQATLDLFRD--------------SGRFYRNAEEMFAEISWVQVMV
Caulobacter   -RHFRQADIDRYNRTMTFEYEKIRDFIILHFKATARSDTPYWDYLR-EMPIPDYLADKIDLFKG--------------SGRVFRENEELFNDTSWFAVFI
Pseud_fl      -TSFDPRLRDAFNAEIVYMFDDCRDFVQAHYFTTSREDTPFWLANRHELRLSDAIQEKVERYKAGLPLTTTSFDDSTYYETFDYEFKNFWLNGNYYCIFA
Burkpyrro     -TSFDPRLTDAFNAEIVYMFDDCRDFVQAHYFATSRDDTPFWLANRHDLRLSDAIKEKVQRYKAGLPLTTTSFDDSTYYETFDYEFKNFWLNGNYYCIFA
Pschloro      -TSFDPRLSDAFNAEIVYMFDDCRDFVQAHYFATSREDTPFWLANRHDLRLSDAIKEKVQRYKAGLPLTTTSFDDSTYYETFDYEFKNFWLNGNYYCIFA
Myxfulvus     -KHIEPVLRDKFNERVSYMVDDVRDFIVLHFCTSPRTDTPYWKANQNELKLPDTLKEVLELQRAGIPIRRSYHTNDALYSSFEAGFDRFWTNSNFQSIFA
Shewanella    NIDDVDYLAKRFNDTVGYAWERVVEFAKLHYCLSDRTDSAFWLDNQREETISEPLKERLNMWKSFSP-----------IAEDFPSKFEVFNLDNYLYVLY
Bdellovibrio  -TLDDQISITGINNEIAVTWDTFRWFVGMHYKFNKKLDTPFWKHCREKTNIGHAQTIVNLFNQRPPLSAG----NFGSNSQFTATEALVFNSYSYDTLIF
P-SSM2        ------NSIDECNEIVNGIFDNIFDYVQAHYLVK-KEDTPFWREVKNDLKITPSLQNLLDKWKKRFP-----------LVGDVFCRWGMFSEVNYIIILQ
Bordetella    ----------------------------------------------------------------------------------------------------
Consensus

                     510       520       530       540       550       560       570
              ....|....|....|....|....|....|....|....|....|....|....|....|....|....|...
Xanaxon       GQGILPQGYHPLVDQVPD--RDAEGFLASVAQTISHCVDVMPSHQQFIDRYCKAATLP---------------
Xancamp       GQGILPQGYHPLVDQVPD--TDAEGFLASVAQTISHCVDVMPTHQQFIDRYCRADRVR---------------
Caulobacter   GQGIYPESYDPMVDCMDE--TLFKARMAEIKSVIARSAEVMPGHMDFIRENCAAEG-----------------
Pseud_fl      GLGMLPDRSLPLLQHRPESIQKAEAMFASIRREAERLRTSLPTNYDYLRSRRDGDAQLSRNQHGPTLAAQERQ
Burkpyrro     GLGMLPDRSLPLLRHRPESIDKAEAMFAIRRREAERLRTSLPTNYDYLRSLRDGDAGLSRSQPGSTLAAPEIL
Pschloro      GLGMLPDRSLPLLQHRPESIEKAEAMFASIRRETERLRTSLPTNYDYLRSLRDGDTGLSHSSSGPNVEAQETL
Myxfulvus     GVGYLPRQPMPLLHSRPDILAEAERTFDDLRQKTAQMMSRLPSQYDYLTSLYARGSRAREEERQRAG------
Shewanella    GMKYPTQPHDRGLVAKAN----LNAYMSNMSNVKRQMLEGLPEHRELIDKICTYGLQPI--------------
Bdellovibrio  GQKEVYKPLAAPEMSKEEYFGKLKSYQELTSRSLSLFELFKGDHLFEDEILEQLFNDTDTWIMEADV------
P-SSM2        GLKWFDSEKVMQEYKNIEHIPLIDWKDTSDRCLSMGHKNFIKELVRIHNLNKEN-------------------
Bordetella    -------------------------------------------------------------------------
Consensus
```
