# Supplementary material for: Three Prochlorococcus Cyanophage Genomes: Signature Features and Ecological Interpretations
Source: PLoS Biol. 2005 Apr 19;3(5):e144. doi: 10.1371/journal.pbio.0030144 (PMC1079782; doi:10.1371/journal.pbio.0030144)
Supplement: Supplementary file 6 [file pbio.0030144.sg006.pdf]

Supplementary Figure 6: Hemagglutinin neuraminidase alignment

```
                           10        20        30        40        50        60        70        80        90       100       110
                   ....|....|....|....|....|....|....|....|....|....|....|....|....|....|....|....|....|....|....|....|....|....|
Gparamyxovirus     -------------MDRAVNRVVLENEEREAKNTWRLVFRIAVLLLMVMTLAISAAALAYSTGASTPHDLAGISTVISKTEDKVTS-----LLSSRQDVIDRIYKQVALES
Newcastle virus    -------------MDRAVSQVALENDEREAKNTWRLIFRIAILFLTVVTLAISVASLLYSMGASTPSDLVGIPTRISRAEEKITS-----TLGSNQDVVDRIYKQVALES
APMV_6             ---------MASSSDMRQSQATLYEGDPNSKRTWRTVYRVITILLDITVLCVGIVAVVRMSTITTKDIDNSISSSITSLSADYQP-----IWSDTHQKVNSIFKEVGITI
HPIV_2             -------------MEDYSNLS----LKSIPKRTCRIIFRTATILGICTLIVLCSSILHEIIHLDVSSGLMDSDDSQQGIIQPIIE-----SLKSLIALANQILYNVAIII
Mumps virus        -----MEPSKLFTMSDNATFAPGPVINAADKKTFRTCFRILVLSVQAVTLILVIVTLGELVRMINDQGLSNQLSSIADKIRESAT-----MIASAVGVMNQVIHGVTVSL
BPIV_3             MEYWKHTNSINNTNNETETARGKHSSKVTNIIMYTFWTITLTILSVIFIMILTNLIQENNHNKLMLQEIRKEFAAIDTKIQRTSDDIGTSIQSGINTRLLTIQSHVQNYI
HPIV_3             MEYWKHTNHGKDAGNELETSMATHNNKLTNKIIYILWTIILVLLSIVFIIVLINSINSEKVHNSLLQEINNEFMEITEKIQMASDNTNDLIQSGVNTRLLTIQSHVQNYI
Sendai virus       MDGDRSKRDSYWSTSPGGSTTKLVSDSERSGKVDTWLLILAFTQWALSIATVIICIVIAARQGYSMERYSMTVEALNTSNKEVKESLTSLIRQEVITRAANIQSSVQTGI
HPIV_1             MAEKGKTNSSYWSTTRNDNSTVNTHINTPAGRTHIWLLIATTMHTVLSFIIMILCIDLIIKQDTCMKTNIMTVSSMNESAKIIKETITELIRQEVISRTINIQSSVQSGI
Ferdelance virus   -----MDPKSYYCN---EDLRSDGGEKSPGGDLYKGIILVSTVISLIIAIISLAFIIDNKINIQSLDPLRGLEDSYLVPIKDKSESISQDIQEGIFPRLNLITAATTTI
ProMED4            --------------------------------------------------------------------------------------------------------------
P-SSM4             --------------------------------------------------------------------------------------------------------------
Consensus

                          120       130       140       150       160       170       180       190       200       210       220
                   ....|....|....|....|....|....|....|....|....|....|....|....|....|....|....|....|....|....|....|....|....|....|
Gparamyxovirus     PLALLNTESIIMNAIPSLSYQINGAANNSGCG--APVHDP--DYIGGIGKELIVGDISDVTSFYPSAYQEHLNFIPAPTTGSGCTRIPSFDMSTTHYCYTHNVILSGCRD
Newcastle virus    PLALLNTETTIMNAITSLSYQINGAANNSGWG--APIHDP--DYIGGIGKELIVDDASDVTSFYPSAFQEHLNFIPAPTTGSGCTRIPSFDMSATHYCYTHNVILSGCRD
APMV_6             PVTLDKMQVEMGTAVNIITDAVRQLQGVNGSAGFSITNSP--EYSGGIDTLIYPLNSLNGKALAVSDLLEHPSFIPAPTTSHGCTRIPTFHLGYRHWCYSHNTIEPGCHD
HPIV_2             PLKIDSIETVIFSALKDMHTGSMSNTNCTPGN--LLLHDA--AYINGINKFLVLKSYNGTPKYG--PLLNIPSFIPSATSPNGCTRIPSFSLIKTHWCYTHNVMLGDCLD
Mumps virus        PLQIEGNQNQLLSTLATICTGKKQVSNCSTNI--PLVNDL--RFINGINKFIIEDYATHDFSIG--HPLNMPSFIPTATSPNGCTRIPSFSLGKTHWCYTHNVINANCKD
BPIV_3             PLSLTQQMSDLRKFINDLTNKREHQ--EVPI--QRMTHDRGIEPLNPNKFWRCTSGNPSLTSSPKIRLIPGPGLLATSTTVNGCIRIPSLVINHLIYAYTSNLITQGCQD
HPIV_3             PISLTQQMSDLRKFISEITIRNDNQ--EVPQ--QRITHDVGIKPLNPDDFWRCTSGLPFLMRNPKIRLMPGPGLLAMPTTVDGCVRTPSLIINDLIYAYTSNLITRGCQD
Sendai virus       PVLLNKNSRDVIRLIEKSCNRQELT--QLCDSTIAVHHAEGIAPLEPHSFWRCPAGEPYLSSDPEVSLLPGPSLLSGSTTISGCVRLPSLSIGEAIYAYSSNLITQGCAD
HPIV_1             PILLNKQSRDLTQLIEKSCNRQELA--QICENTIAIHHADGISPLDPHDFWRCPVGEPLLSNNPNISLLPGPSLLSGSTTISGCVRLPSLSIGDAIYAYSSNLITQGCAD
Ferdelance virus   PRSIAIQTKDLSDLIMNRCYPSVVNNDTSCDVLAGAIHSNLFSQLDPSTYWTCSSGTP--TMNQTVKLLPDNSQIPGSTYSTGCVRIPTFSLGSMIYSYSHNVIYEGCND
ProMED4            ----------------------------------------------------------------------------------------------------MKLRFFPTTIFR
P-SSM4             ---------------------------------------------------------------------------------------------------VSDVLFRKHRVFR
Consensus

                          230       240       250       260       270       280       290       300       310       320       330
                   ....|....|....|....|....|....|....|....|....|....|....|....|....|....|....|....|....|....|....|....|....|....|
Gparamyxovirus     HSHSHQYLALGVLRTSATGRVFFSTLRSINLDDTQNRKSCSVSATPLGCDMLCSKVTGTEEEDYKSVAPTSMVHGRLGFDGQYHEKDLDTT--VLFKDWVANYPGAGGGS
Newcastle virus    HSHSYQYLALGVLRTSATGRVFFSTLRSINLDDTQNRKSCSVSATPLGCDMLCSKATETEEEDYNSAVPTRMVHGRLGFDGQYHEKDLDVT--TLFGDWVANYPGVGGGS
APMV_6             AGESIMYVSMGAVGVGHRGKPVFTTSAATILDDGRNRKSCNIIANPNGCDVLCSLVKQTENEDYADPTPTPMIHGRLHFNGTYTESELNPG--LFNNHWVAQYPAVGSGV
HPIV_2             FTTSNQYLAMGIIQQSAAAFPIFRTMKTIYLSDGINRKSCSVTAIPGGCVLYCYVATRSEKEDYATTDLAELRLAFYYYNDTFIERVISLP--NTTGQWATINPAVGSGI
Mumps virus        HTSSNQYISMGILVQTASGYPMFKTLKIQYLSDGLNRKSCSIATVPDGCAMYCYVSTQLETDDYAGSSPPTQKLTLLFYNDTVTERTISPT--GLEGNWATLVPGVGSGI
BPIV_3             IGKSYQVLQIGIITINSDLVPDLNPRVTHTFNIDDNRRSCSLALLNTDVYQLCSTPKVDERSDYASTGIEDIVLDIVTNNGLIITTRFTNNNITFDKPYAALYPSVGPGI
HPIV_3             IGKSYQVLQVGIITVNSDLVPDLNPRFSHTFNINDNRKSCSLALLNTDVYQLCSTPKVDERSDYASSGIEDIVLDIVNYDGSISTTRFKNNNISFDQPYAALYPSVGPGI
Sendai virus       IGKSYQVLQLGYISLNSDMFPDLNPVVSHTYDINDNRKSCSVVATGTRGYQLCSMPIVDERTDYSSDGIEDLVLDILDLKGRTKSHRYSNSEIDLDHPFSALYPSVGSGI
HPIV_1             IGKSYQVLQLGYISLNSDMYPDLNPVISHTYDINDNRKSCSVIAAGTRGYQLCSLPTVNETTDYSSEGIEDLVFDILDLKGKTKSHRYKNEDITFDHPFSAMYPSVGSGI
Ferdelance virus   HSKSSQYWQLGYISTSKTGEPLQQVSRTLTLNNGLNRKSCSTVAQGRGAYLLCTNVVEDERTDYSTEGIQDLTLDYIDIFGAERSYRYTNNEVDLDRPYAALYPSVGSGT
ProMED4            ETPKVTFFDAGLEASNGCDVVIHSEEAISPPDDFK-DEQYYVHNHQSDHNLVIAGERTFVLINPAWDEPHHVIYLNRSMGALEIPIGTYHRSISGKEGSIVLNQPTRDKF
P-SSM4             ETQDVIFFDISVEESNASDLVVHTGAAISPPNDLVGAKQFYIHYHQTDFNRVVQGERQFELVNFDWKYPYHIVHLNRSSGALVIPTKTFHRSISGEEGSIVINQSSRTEG
Consensus             .         .                            .      ..                              :                                .

                          340       350       360       370       380       390       400       410       420       430       440
                   ....|....|....|....|....|....|....|....|....|....|....|....|....|....|....|....|....|....|....|....|....|....|
Gparamyxovirus     FIDDRVWFPVYGGLKPNSPSDTAQEGKYVIYKRHNNTCPDEQDYQIRMAKFSYKPGRFGGKRVQQAILSIKVSTSLGKDPVLTIPPNTITLMGAEGRILTVGTSHFLYQR
Newcastle virus    FIDSRVWFSVYGGLKPNSPSDTVQEGKYVIYKRYNDTCPDEQDYQIRMAKSSYKPGRFGGKRIQQAILSIKVSTSLGEDPVLTVPPNTVTLMGAEGRILTVGTSHFLYQR
APMV_6             VSHGKLFFPLYGGISPKSKLFNELKSFAYSTHHAELKCENLTERQKKDLYNAYRPGKIAGSLWAQGVVTCNLTN--LADCKVAIANTSTMMMAAEGRLQLVQDRIVFYQR
HPIV_2             YHLGFILFPVYGGLISGTPSYNKQSSRYFIPKHPNITCAGNSSEQAAAARSSYVIRYHSNRLIQSAVLICPLSDMHTARCNLVMFNNSQVMMGAEGRLYVIDNNLYYYQR
Mumps virus        YFENKLIFPAYGGVLPNSSLGVKSAREFFRPVNPYNPCSGPQQDLDQRALRSYFPSYFSNRRVQSAFLVCAWNQILVTNCELVVPSNNQTLMGAEGRVLLINNRLLYYQR
BPIV_3             YYKDKVIFLGYGGLEHE------------ENGDVICNTTGCPGKTQRDCNQASYSPWFSNRRMVNSIIVVDKGIDATFSLRVWTIPMSQNYWGSEGRLLLLGDRIYIYTR
HPIV_3             YYKGKIIFLGYGGLEHP------------INENVICNTTECPGKTQRDCNQASYSPWFSDRRMVNSIIVVDKGLNSIPKLKVWTISMRQNYWGSEGRLILLGNKIYIYTR
Sendai virus       ATEGSLIFLGYGGLTTP------------LQGDTKCRIQGCQQVSQDTCNEALKITWLGGKQVVSVLIQVNDYLSERPRIRVTTIPITQNYLGAEGRLLKLGDQVYIYTR
HPIV_1             KIENTLIFLGYGGLTTP------------LQGDTKCVINRCTNVNQSVCNDALKITWLKKRQVVNVLIRINNYLSDRPKIVVETIPITQNYLGAEGRLLKLGKKIYIYTR
Ferdelance virus   VYNDRILFLGYGGLMTP------------YGDQAMCQAPECTSATQEGCNSNQLIGYFSGRQIVNCIIEIITVGTEKPIIRVRTIPNSQVWLGAEGRIQTLGGVLYLYIR
ProMED4            FDPAKEFIPQK--------------------LDQISLIKARKTPPVYWVWENDQIKKVGFNPIEREIKSLT---------------------------------------
P-SSM4             FDHNKEFIP--------------------------VSAAENKKLYEILKHEKPVVHTLGE-------------------------------------------------
Consensus                :

                          450       460       470       480       490       500       510       520       530       540       550
                   ....|....|....|....|....|....|....|....|....|....|....|....|....|....|....|....|....|....|....|....|....|....|
Gparamyxovirus     GSSYFSPALLYPMTVNNKT------ATLHSPYTFNAFTRPGSVP--CQASARCPNSCITGVYTDPYPLIFHRNHTLRGVFG------TMLDDEQARLNPVSAVFDNISRS
Newcastle virus    GSSYFSPALLYPMTVSNKT------ATLHSPYTFNAFTRPGSIP--CQASARCPNSCVTGVYTDPYPLIFYRNHTLRGVFG------TMLDGEQARLNPASAVFDSTSRS
APMV_6             SSSWWPVLIYYDIPISDLISADHLEIVNWTPYPQSKFPRPTWTKGVCEKPAICPAVCVTGVYQDVWVVSIGSQSNETVVVG------GYLDAAAARQDPWIAAANQYNWL
HPIV_2             SSSWWSASLFYRINTDFSKGIP--PIIEAQWVPSYQVPRPGVMP--CNATSFCPANCITGVYADVWPLNDPEPTSQNALNPNYRFAGAFLRNESNRTNPTFYTASASALL
Mumps virus        STSWWPYELLYEISFTFTNSGQ--SSVNMSWIPIYSFTRPGSGN--CSGENVCPTACVSGVYLDPWPLTP--YSHQSGINRNFYFTGALNSSTTRVNPTLYVSALNNLK
BPIV_3             STSWHSKLQLGVIDISDYT------NIRINWTWHNVLSRPGNDE--CPWGHSCPDGCITGVYTDAYPLNPSGSVVSSVILD----------SQKSRENPIITYSTATNRI
HPIV_3             STSWHSKLQLGIIDITDYS------DIRIKWTWHNVLSRPGNNE--CPWGHSCPNGCITGVYTDAYPLNPTGSIVSSVILD----------SQKSRVNPVITYSTATERV
Sendai virus       SSGWHSQLQIGVLDVSHP--------LTISWTPHEALSRPGNED--CNWYNTCPKECISGVYTDAYPLSPDAANVATVTLY----------ANTSRVNPTIMYSNTTNII
HPIV_1             SSGWHSNLQIGSLDINNP--------MTIKWAPHEVLSRPGNQD--CNWYNRCPRECISGVYTDAYPLSPDAVNVATTTLY----------ANTSRVNPTIMYSNTSEII
Ferdelance virus   SSGWHALAQTGIILTLDP--------IRISWIVNTGYSRPGNGP--CSASSRCPAQCITGVYTDIFPLSQNYGYLATVTLL----------SGVDRVNPVISYGTSTGRV
ProMED4            --------------------------------------------------------------------------------------------------------------
P-SSM4             --------------------------------------------------------------------------------------------------------------
Consensus

                          560       570       580       590       600       610       620       630
                   ....|....|....|....|....|....|....|....|....|....|....|....|....|....|....|....|....|....
Gparamyxovirus     RVTRVSSSSTKAAYTTSTCFKVVKTNKAYCLSIAEISNTLFGEFRIVPLLVEILKDNRV------------------------------
Newcastle virus    RITRVSSSSIKAAYTTSTCFKVVKTNKTYCLSIAEISNTLFGEFRIVPLLVEIKDDGVREARSG-------------------------
APMV_6             VRRRLFTSQTEAAYSSTTCFRNTKQDRVFCLTIMEVTDNLLGDWRIAPLLYEVTVADKQQGNRNYAPMGRMGTDKFQYYTPGDKYTPQH
HPIV_2             NTTGFNNTNHKAAYTSSTCFKNTGTQKIYCLIIIEMGSSLLGEFQIIPFLRELIP----------------------------------
Mumps virus        VLAPYGNQGLFASYTTTTCFQDTGDASVYCVYIMELASNIVGEFQILPVLTRLTIT---------------------------------
BPIV_3             NELAIYNRTLPAAYTTTNCITHYDKG--YCFHIVEINHRSLNTFQPMLFKTEVPKNCS-------------------------------
HPIV_3             NELAIRNRTLSAGYTTTSCITHYNKG--YCFHIVEINQKSSNTFQPMLFKTEIPKSCSQS-----------------------------
Sendai virus       NMLRIKDVQLEAAYTTTSCITHFGKG--YCFHIIEINQKSLNTLQPMLFKTSIPKLCKAES----------------------------
HPIV_1             NMLRLKNVQLEAAYTTTSCITHFGKG--YCFHIVEINQASLNTLQPMLFKTSIPKICKITS----------------------------
Ferdelance virus   ADSQLTSSSQVAAYTTTTCFTFNQKG--YCYHIIELSPATLGIFQPVLVVTEIPKICS-------------------------------
ProMED4            -----------------------------------------------------------------------------------------
P-SSM4             -----------------------------------------------------------------------------------------
Consensus
```
